# Mucoadhesive, antioxidant, and lubricant catechol-functionalized poly(phosphobetaine) as biomaterial nanotherapeutics for treating ocular dryness

**DOI:** 10.1186/s12951-024-02448-x

**Published:** 2024-04-08

**Authors:** Hoang Linh Bui, Yun-Han Su, Chia-Jung Yang, Chun-Jen Huang, Jui-Yang Lai

**Affiliations:** 1 Department of Biomedical Sciences and Engineering, National Central University, Taoyuan, 32023 Taiwan; 2 Department of Biomedical Engineering, Chang Gung University, Taoyuan, 33302 Taiwan; 3 Department of Chemical and Materials Engineering, National Central University, Taoyuan, 32023 Taiwan; 4 R&D Center for Membrane Technology, Chung Yuan Christian University, Taoyuan, 32023 Taiwan; 5 NCU-Covestro Research Center, National Central University, Taoyuan, 32023 Taiwan; 6 Department of Ophthalmology, Chang Gung Memorial Hospital, Linkou, 33305 Taoyuan Taiwan; 7 Department of Materials Engineering, Ming Chi University of Technology, New Taipei City, 24301 Taiwan; 8 Research Center for Chinese Herbal Medicine, College of Human Ecology, Chang Gung University of Science and Technology, Taoyuan, 33303 Taiwan; 9 Center for Biomedical Engineering, Chang Gung University, Taoyuan, 33302 Taiwan

**Keywords:** Multifunctional polyzwitterion, Catechol, Poly(phosphobetaine), Biomaterial nanotherapeutics, Ocular dryness

## Abstract

Dry eye disease (DED) is associated with ocular hyperosmolarity and inflammation. The marketed topical eye drops for DED treatment often lack bioavailability and precorneal residence time. In this study, we investigated catechol-functionalized polyzwitterion p(MPC-*co*-DMA), composed of 2-methacryloyloxyethyl phosphorylcholine (MPC) and dopamine methacrylamide (DMA) monomers, as potential topical nanotherapeutics for DED. The copolymers were synthesized via random free-radical copolymerization, producing different proportions of catecholic functionalization. All as-prepared polymer compositions displayed good ocular biocompatibility. At a feeding ratio of 1:1, p(MPC_1_-*co*-DMA_1_) can facilitate a robust mucoadhesion via Michael addition and/or Schiff base reaction, thus prolonging ocular residence time after 4 days of topical instillation. The hydration lubrication of MPC and radical-scavenging DMA endow the nano-agent to ease tear-film hyperosmolarity and corneal inflammation. A single dose of p(MPC_1_-*co*-DMA_1_) (1 mg/mL) after 4 days post-instillation can protect the cornea against reactive oxygen species, inhibiting cell apoptosis and the over-expression of pro-inflammatory factors (IL-6 and TNF-α). In clinical assessment, DED-induced rabbit eyes receiving p(MPC_1_-*co*-DMA_1_) could increase lacrimal fluid secretion by 5-fold higher than cyclosporine A. The catechol-functionalized polyzwitterion with enhanced lubricity, mucoadhesion, and anti-oxidation/anti-inflammation properties has shown high promise as a bioactive eye drop formulation for treating DED.

**Supplementary Information:**

The online version contains supplementary material available at 10.1186/s12951-024-02448-x.

## Graphical abstract

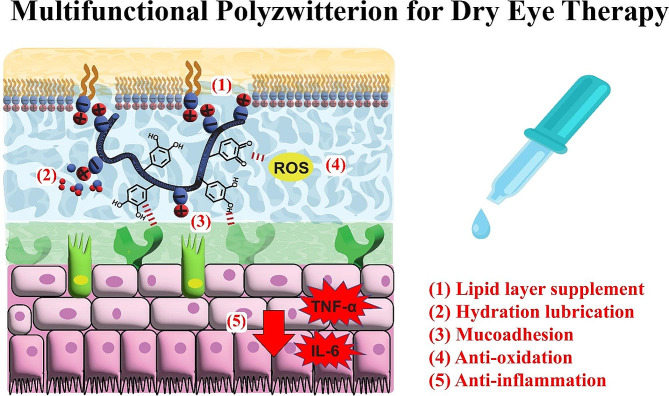

## Introduction

Dry eye disease (DED) is a common multifactorial ocular condition classified as one of the most reasons for patients to seek eye care [1, 2]. By definition, the symptoms of DED patients include a decrease in tear film homeostasis and ocular surface inflammation [3]. DED is also identified clinically by measuring tear fluid volume, tear film break-up time, corneal staining and tear osmolarity [4]. In Taiwan, the incidence of DED has increased by approximately three times from 1.46 to 4.26 (per 1000 population) over fifteen years (from 2001 to 2015). Dry eye symptoms are more common in the older population [5], but recent epidemiological studies show an increase in the prevalence of DED in visual display workers (mainly young adults) within the social distancing period during the coronavirus (COVID-19) pandemic [6]. In addition, the perseverant usage of 3 C (computer, communication, and consumer electronic products) products and low relative humidity conditions (air travel, indoor environment, and air pollution) could also contribute to increased DED frequency in the population [7]. On the basis of daily life, a loss of visual function and inflammation-associated discomfort and irritation can cause a significant burden on DED patients. Hence, DED has become the subject of many studies that pertain to the cause and treatment of DED [4, 8, 9].

Topical ocular administration has remained the most preferred drug delivery route owing to its ease of operation, safety, and affordability [10]. However, topical drops of medications are prevented from reaching targeted ocular tissue by innate anatomical and physiological barriers [11]. This ultimately leads to a reduction in bioavailability of drug and its intrinsic pharmacological activity. Additionally, the variations in pharmacodynamics and pharmacokinetics of multiple doses of medications created challenges for clinicians to avoid the occurrence of adverse events [12, 13]. Furthermore, numerous eye illnesses (including DED) are generally chronic, leading to frequent and extensive medication treatments. Therefore, it is necessary to develop novel nanotherapeutics to overcome the low bioavailability of topical administration.

Zwitterionic polymer is a class of macromolecule that contains an equimolar number of cationic and anionic functional groups [14]. Owing to their distinct chemical structure, polyzwitterions can exhibit superior hydrophilicity, binding up to eight water molecules via electrostatic interaction [15]. While zwitterionic materials have been well-recognized for non-fouling surface modification, the polymers also offer a vast contribution to therapeutic applications, including maintenance of bioactivity of nano- and microscale complexes in vivo [16, 17], drug solubility enhancement and delivery [18, 19]. Among the zwitterion family, poly(2-methacryloyloxyethyl phosphorylcholine) (p(MPC)), containing an anionic phosphate group, resembles the most as the phosphatidylcholine lipid bilayer found in human cell membrane [20]. Moreover, p(MPC) and MPC monomers also exhibit high biocompatibility in both in vitro and in vivo models [21, 22]. Importantly, Food and Drug Administration (FDA) and Pharmaceuticals and Medical Devices Agency (PMDA) have so far given their approval for MPC-containing contact lenses [23, 24]. Owing to its biocompatibility, p(MPC) has been recently applied once daily for 5 days to promote recovery in DED rabbit model, showing an enhancement in lacrimal fluid volume and an improvement in the tear film break-up levels [25]. In addition, p(MPC) has been well-recognized as a lubricating agent [26, 27], which could provide osmoprotectant from tear desiccation. However, ophthalmic drops that are composed of p(MPC) polymers alone have several shortcomings. MPC homopolymer with its hydrophilicity and neutral charge showed limitation in interfacial retention [15, 28]. Accordingly, it is speculated that p(MPC) used in topical eye drop can easily be removed due to tear dilution and the clearance action caused by the lacrimal drainage system. On the other hand, polymeric materials have been implemented as in situ gel systems and ophthalmic devices for DED treatment [29]. These hydrogels with enhanced viscosity can facilitate corneal retention and are generally regarded as safe, making them available on the market and undergoing clinical trials [29]. However, regulating the drug release pattern in hydrogel systems is challenging, and inadequate management of initial burst release could lead to severe ocular adverse reactions.

In the development of polymeric therapeutics for DED, two ideal properties can be summed up as follows: (1) Considering DED vicious cycle represents the interplays among oxidative stress, inflammation, tear-film hyperosmolarity, cell apoptosis, and tear film deuteriation [30–32], the development of nanobiomaterial-based formulation with a comprehensive treatment is highly desirable; (2) To avoid static, dynamic, and metabolic barriers of the eye [4], polymers should be equipped with robust bio-adhesive properties for the improvement of therapeutic effect along with their precorneal residence time. Based on the two mentioned categories, conjugating dopamine (DA) with p(MPC) may render an effective combination treatment for ocular dryness. DA was inspired by 3,4-dihydroxy-L-phenylalanine (DOPA), a distinct catechol-containing amino acid from marine mussels [33]. The catechol moieties in DA and DA-functionalized polymers exhibit a strong interfacial interaction with biological samples. For example, Lee and colleagues found that catechol-functionalized chitosan showed mucoadhesive properties in the intestine specimen [34]. Wu and colleagues introduced a dual-crosslinked interpenetrate network using DA as dynamic crosslinks, which achieved robust skin adhesion (∼ 20 kPa) beyond 1 month [35]. For therapeutic purposes, polydopamine (PDA), which is a polymer configuration of DA, can efficiently quench multiple excessive reactive oxygen and nitrogen species similarly to superoxide dismutase enzyme, and protects ischemic brains from oxidative damage [36]. In addition, Wang and colleagues further demonstrated that PDA not only exhibits radical scavenging properties, but also facilitate anti-inflammatory effects due to the down-regulation of pro-inflammatory cytokines and alleviation of neutrophil infiltration, which decreases lung tissue damage in mice models [37]. Recently, N-(3,4-dihydroxyphenethyl)methacrylamide (DMA), which is a DA-derived polymerizable molecule, has also been used as a radical scavenging and anti-inflammatory agent to treat osteoarthritis [38].

Herein, we demonstrate the potential use of catechol-functionalized polyphosphobetaine (p(MPC-*co*-DMA)) to treat DED via topical administration (Schema 1). The copolymerization of hydrophilic MPC and ROS-scavenging DMA via random copolymerization has synergistic benefits in that it conditions tear film stability and creates anti-inflammatory mechanisms to allow the complete recovery of dry eyes. Additionally, DMA residues also allows wet adhesiveness on the ocular surface to enhance retention time and prolong therapeutic action of the copolymers. As-prepared p(MPC) and p(MPC-*co*-DMA) compositions were characterized using ^1^H nuclear magnetic resonance (^1^H NMR), gel permeation chromatography (GPC), and ultraviolet-visible spectroscopy (UV-VIS). The hydration lubrication properties of synthetic polymers were determined using a standardized friction test. Mucins (glycosylated proteins) are essential components of the ocular surface and are expressed as membrane-bound proteins on corneal and conjunctival epithelial cells or suspended within the mucous layer of tear film [39]. To demonstrate the mucoadhesive properties of the synthetic compounds, the interaction of copolymers with mucin-deposited silicon wafers was evaluated using UV-VIS, water contact angle (WCA) measurement, and X-ray photoelectron spectroscopy (XPS). Additionally, an electrochemical Quartz Crystal Microbalance (eQCM) was also employed to monitor the modes of adhesion for catechol-containing polymer on the mucin layer. For biological activities, p(MPC-*co*-DMA) in this study was evaluated for its antioxidant capability, in vitro biocompatibility, and ROS cytoprotective properties. Finally, the therapeutic potential of the polymers was determined using a 4-day experimental set with a rabbit model of DED. Histological studies, including oxidative stress, cell apoptosis, and cellular inflammation (inflammatory factors including IL-6 and TNF-α) were assessed to validate the therapeutic effect of the copolymers. Overall, the results of this study demonstrate a new approach utilizing polymeric biomaterial nanotherapeutics for DED treatment and provide insight into both physicochemical and biological properties of p(MPC-*co*-DMA) copolymers.

**Scheme 1:**
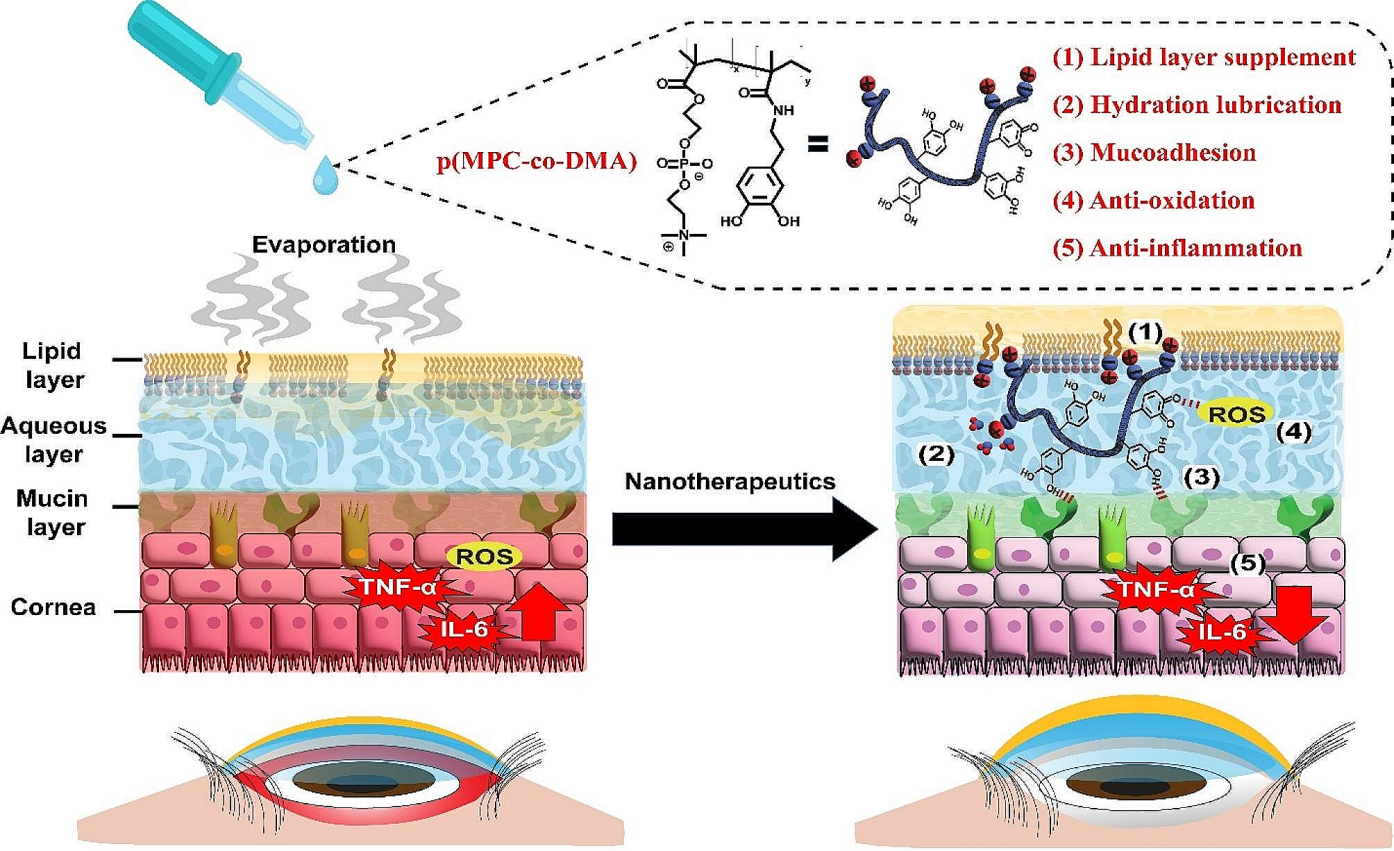
Schematic representations of p(MPC-*co*-DMA) and its therapeutic effects on DED.

## Experimental

### Materials

2-Methacryloyloxyethyl phosphorylcholine (MPC, 97%), dopamine hydrochloride (DA), 2,2’-diphenyl-1-picrylhydrazyl (DPPH), benzalkonium chloride (BAC), methacrylic anhydride, tetrahydrofuran (THF), ethyl acetate, sodium sulfate anhydrous, hexane, 2,2’-Azobis(isobutyronitrile) (AIBN), acetone, ethanol, phosphate buffered saline (PBS), dimethyl sulfoxide (DMSO), and Folin-Ciocalteu reagent were purchased from Sigma-Aldrich (St. Louis, MO). Omega membrane (3 K MWCO) was bought from Pall Corporation. Sodium hydroxide (NaOH) and hydrochloric acid (HCl) were obtained from Thermo Scientific (Waltham, MA). Sodium tetraborate and sodium citrate were acquired from Showa (Tokyo, Japan). Sodium bicarbonate was received from Acros Organics (Belgium). Eagle’s minimum essential medium (MEM) was purchased from Gibco-BRL (Grand Island, NY, USA). Fetal bovine serum (FBS) and the antibiotic/antimycotic (A/A) solution (10,000 U/mL penicillin, 10 mg/mL streptomycin, and 25 µg/mL amphotericin B) were purchased from Biological Industries (Kibbutz Beit Haemek, Israel). A Live/Dead Viability/Cytotoxicity Kit was purchased from Molecular Probes (Eugene, OR, USA). Xylazine hydrochloride was bought from Bayer (Leverkusen, Germany). Tiletamine hydrochloride/zolazepam hydrochloride was received from Virbac (Carros, France). Deionized (DI) water used in all experiments was filtered using a millipore water purification system with a minimum resistivity of 18.3 MΩ·m.

### Synthesis of p(MPC) homopolymer and p(MPC-co-DMA) copolymer

p(MPC) homopolymer and three different compositions of p(MPC-*co*-DMA), containing different molar ratios (MPC: DMA = 1:1, 3:1, and 6:1, respectively), are represented as p(MPC_1_-*co*-DMA_1_), p(MPC_3_-*co*-DMA_1_), and p(MPC_6_-*co*-DMA_1_). The polymers were synthesized using simple thermal-triggered free radical polymerization using AIBN as an initiator and DMSO as a solvent. In detail, for the synthesis of p(MPC) homopolymer, MPC monomer (442.9 mg, 1.50 mmol) and AIBN (4.125 mg, 0.014 mmol) were added to a glass reaction tube and mixed thoroughly with 5 mL of DMSO under sonication. After degassing with argon gas for 30 min, polymerization was allowed to occur at 65 °C in an oil bath for 24 h. After that, a small amount of deionized water was added to dissolve p(MPC) and improve its production yield. The resultant polymer was then dialyzed against deionized water (DI) using a dialysis membrane (8 K MWCO, Spectrum Laboratories) for 3 days with periodic bath changes and lyophilized as a powder. For the routine synthesis of p(MPC-*co*-DMA), DMA (55.3 mg, 0.25 mmol) was mixed with MPC (73.8 mg, 0.25 mmol for 1:1; 221.4 mg, 0.75 mmol for 3:1; and 442.9 mg, 1.5 mmol for 6:1) and AIBN (4.125 mg, 0.014 mmol) in 5 mL of DMSO. The polymerization conditions and purification processes of p(MPC-*co*-DMA) copolymer are similar to those for p(MPC).

### Characterization studies

For the characterization of synthetic products, ^1^H NMR spectra were recorded using a 600-MHz Bruker Ascend spectrometer. UV-VIS absorption spectra were obtained using a UV-1900 spectrophotometer (Shimadzu, Japan). FTIR analysis was performed using IRSpirit spectrometer (Shimadzu, Japan). For a routine sample preparation, a small amount of powdered sample is mixed with a powdered alkali KBr, pressed into a pellet using a pellet die, and placed into a pellet holder for transmission FTIR measurement. The non-functional and functional samples were identified via recording their FTIR spectra in the range of 650–4000 cm^− 1^ with a resolution of 1 cm^− 1^. The molecular weight of p(MPC) and p(MPC-*co*-DMA) copolymers were measured by GPC, using a Viscotek RI detector (VE3580, Malvern). The mobile phase is 0.15 M NaCl solution, containing 0.02 wt% sodium azide. The calibration standard curves were plotted using polyethylene glycol (PEG) (Mw 450 − 120,000 Da). The specific polymer size was achieved using a flow rate of 0.6 mL/min at 40 °C in an A3000-Single-pore GPC/SEC column (300 × 8 mm) and a CLM3023-A guard column (50 × 6 mm). All samples were cleaned of contaminants using 0.22 μm filters before testing.

### Friction measurements

To determine the lubrication properties of the synthesized polymers, pristine silicon tubes and mucin- or polymer-smeared silicon tubes were used for the friction measurement. To produce mucin-coated silicon tube, the substrate was dipped into mucin solution (1 wt% mucin in PBS buffer) for 1 h at 37 °C to generate a mucin layer via passive adsorption. The mucin-coated substrates were then allowed to dry at 30 °C for 45 min, followed by polymer deposition. To produce polymer-coated samples, bare silicon tubes were soaked in polymer solution (1 wt% polymer in PBS buffer) for 1 h at room temperature. The silicon tubes with polymer deposits were then dried in oven at 30 °C for 45 min before the friction test was performed. The coefficient of friction was measured using an universal testing machine (QC-513M1F, Cometech, Taiwan) with a coefficient of friction fixture (QC-117, Cometech, Taiwan). The procedure complies with the ASTM D1894 and ISO 8295 standards. In brief, two silicon tubes (with and without surface modification) were attached to a sled (200 mg) using steel wires. The sled was then pulled using a normal force of 2 N to acchieve a sliding velocity of 150 mm/min across a silicon sheet with the hardness of 60 A under DI water. The drag displacement was 130 mm. The forces that were required to pull the sled (static friction) and to maintain motion (kinetic friction) were interpreted as coefficient of friction (COF). The sliding cycles were repeated three times to determine the stability of the coating. Six coated silicon tubes (mucin or polymer solution) were used for three independent experimental sets (two tubes for each coating condition) to calculate the average value for COF.

### Mucoadhesive studies

Mucin solution (1 wt%) was mixed with synthesized polymers (1 wt%) with different compositions in PBS at pH 7.4. Absorbance was measured immediately after complexation using UV-VIS. In WCA and XPS analysis, mucin and mucin/P coatings that were deposited on silicon wafers. The deposition process for the coating is similar to that for the friction test. XPS spectra in P2p, N1s, C1s, and O1s core-level regions were measured using a Sigma Probe spectrometer (Thermo VG Scientific) with a monochromatic aluminum anode X-ray source (Kα = 1486.6 eV). A contact angle goniometer (Phoenix mini, Korea) was used to determine WCA and the static angle values at solid-liquid interfaces. A 5 µL droplet was dropped using a micro-syringe, and measurements were performed in triplicate at random locations on each sample.

eQCM data was recorded using a Gamry eQCM 10 M quartz crystal microbalance, Gamry Instruments. All of the eQCM experiments were performed in a three-electrode non-thermostatic cell at room temperature. The working electrode was an Au-coated eQCM chip (AT-cut, basic frequency 10 MHz, 971-00006, Gamry Instruments). A continuous flow regime was acchieved using a eQCM flow cell kit (ALS, cat. No. 012026, Tokyo, Japan) and a peristaltic pump (Longer Precision Pump Co., Model BT100-2 J). The Sauerbrey equation is used to calculate the mass density of adsorbed molecules using the quartz oscillation frequency as follows:

$${\Delta }\text{f}=\frac{2{{f}_{0}}^{2}}{\text{A}\sqrt{{{\uprho }}_{\text{q}}.{{\upmu }}_{q} }}{\Delta }\text{m}$$

where Δf represents the frequency shift, f_0_ is the basic oscillation frequency of quartz (Hz), Δm is the change in surface mass, A is the electrode area (0.2047 cm^2^), µ_q_ is the shear modulus (2.95 × 10^11^ dyn/cm^2^) and ρ_q_ is the density of quartz (2.65 g cm^− 3^).

Before each set of experiments, an eQCM chip was cleaned with glass detergent for 10 min and sonicated, followed by rinsing with DI water and 95% ethanol. The quartz crystal was then treated with oxygen plasma for 10 min. The process for mucin coating was adapted from the method of other study with minor modifications [40]: mucin solution (0.025 mg/mL) was injected at a flow rate of 60 µL/min until the value of Δf stabilized. The unbound mucins were then removed by rinsing with PBS buffer for 10 min. The polymer solution (100 mg/mL) was then injected for 30 min, and unbound polymers were removed by rinsing with buffer for another 10 min.

### In vitro biocompatibility tests

Statens Seruminstitut rabbit cornea (SIRC) cells (BCRC no. 60,093) were purchased from the Bioresource Collection and Research Center (Hsinchu, Taiwan, ROC). The SIRC cells were grown in MEM that is supplemented with 10% FBS and 1% A/A solution. Cultures were incubated in a humidified atmosphere of 5% CO_2_ at 37 °C. The medium was replaced twice a week. The biocompatibility of polymers was measured using MTS, live/dead, and comet assays (please see Supporting Information for more details).

### Antioxidant and anti-inflammatory activity studies

The free radical scavenging capacity of p(MPC) and different p(MPC-*co*-DMA) formulations was assessed using DPPH assay. 12.5 mL of ethanol solution with 250 mg of DPPH powder was mixed with an equal volume of p(MPC-*co*-DMA) solution. A UV-VIS spectrophotometer was used to measure the absorbance of the prepared solution at 517 nm, followed by a 30-min incubation period at 25 °C. The DPPH scavenging activity (%) is calculated as ((A_0_ - A_1_)/A_0_) × 100, where A_0_ represents the absorbance of a blank DPPH solution under identical reaction conditions without synthetic polymer samples and A_1_ represents the absorbance of DPPH solution in which polymer samples are present. The results are expressed as the mean for five different measurements. The total phenolic content in the various p(MPC-*co*-DMA) samples was measured using Folin-Ciocalteu reagent and DMA as a standard. Sodium carbonate (1.5 mL, 20%) and Folin-Ciocalteu reagent (1.0 mL) were diluted using the different p(MPC-*co*-DMA) formulations. A UV-VIS absorption spectrometer was used to determine the mixture’s absorbance at 760 nm after incubation for 2 h at room temperature. The results of five separate runs were used to calculate an average value.

The intracellular buildup of ROS was measured as the oxidative-induced conversion of cell-permeable 2’-7’-dichlorodihydrofluorescein diacetate (DCFH-DA) (Molecular Probes) to fluorescent 2’-7’-dichlorofluorescein (DCF). 10 µM DCFH-DA solutions were incubated in culture wells with the SIRC cells for 1 h at 37 °C, and the cells were then rinsed three times with PBS. The DCF fluorescence imaging was used to measure excitation at 488 nm and emission at 525 nm using a fluorescence microscope (Axiovert 200 M; Carl Zeiss, Oberkochen, Germany). The intracellular overexpression of calcium was measured using Fura-2 AM (Molecular Probes) as a Ca^2+^-sensitive fluorescent marker. The SIRC cells were co-incubated with 5 µM Fura-2 AM solutions at 37 °C for 1 h and then rinsed three times with PBS. Fluorescence imaging used excitation at 340 nm and emission at 510 nm using a fluorescence microscope (Carl Zeiss). To determine intracellular calcium, cells were collected and resuspended in N-2-hydroxyethylpiperazine-N’-2-ethanesulfonic acid (HEPES)-buffered saline solution (containing 132 mM NaCl, 2 mM CaCl_2_, 3 mM KCl, 10 mM glucose and 10 mM HEPES, pH 7.4). ROS and Ca^2+^ assays used a multimode microplate reader (BioTek Instruments, Winooski, VT, USA) to determine the variation in the intensity of fluorescence. Each experiment was performed four times and the results for four separate runs were used to calculate an average value. Cells that were exposed to 0 (Ctrl group) or 200 (HP group) µM H_2_O_2_ for 24 h with no polymer pre-treatment were used for comparison.

In anti-inflammatory studies, the SIRC cells at a density of 10^5^ cells/well were seeded in a 24-well plate and incubated in MEM medium for h12 to allow cells to attach. Lipopolysaccharide (LPS) (1.0 µg/mL) was used to trigger the inflammation of SIRC cultures. After 1 h of incubation, various p(MPC-*co*-DMA) samples were added to the wells, and the supernatant was collected after 24 h. Each resultant solution was studied using an IL-6 and TNF-α ELISA Quantikine kit (R&D Systems, Minneapolis, MN, USA). Five independent runs were performed, and the results of each were used to calculate an average value.

### Corneal retention studies

All animals used in in vivo studies were approved by the Institutional Animal Care and Use Committee of Chang Gung University (Approval Number: CGU111-080). The procedures comply with the ARVO Statement for the Use of Animals in Ophthalmic and Vision Research. Twenty-four adult New Zealand white rabbits (National Laboratory Animal Breeding and Research Center, Taipei, Taiwan, ROC) (16–20 weeks old and weighing 3.0–3.5 kg) were used for a corneal retention study. The rabbits were intramuscularly anesthetized with 2.5 mg per kg body weight of a 1:1 mixture of tiletamine hydrochloride/zolazepam hydrochloride and 1 mg per kg body weight of xylazine hydrochloride. For the four test groups of animals (six rabbits/group), corneas that were treated with p(MPC) and different compositions of p(MPC-*co*-DMA) were harvested at h12 and 4 days postoperatively, and analyzed using scanning electron microscopy/energy dispersive spectroscopy (SEM/EDS) (Oxford Instruments, Concord, MA, USA). The elemental distribution was measured using X-ray elemental mapping. Data was acquired using an accelerating voltage of 10 kV. Measurement of the amount of elemental phosphorus (P) in corneal specimens used inductively coupled plasma optical emission spectroscopy (ICP-OES). All experiment sets involved six independent runs and the results were used to calculate an average value.

### Therapeutic efficacy studies

A DED-induced model (DED group) was produced by topical administering 0.15% BAC twice daily for 14 days. For the therapeutic efficacy tests, thirty-six dry-eye rabbits were divided into one control and five test groups, each of which had six animals. The control (Ctrl) group was topically treated with a single dose of 50 µL of artificial tear solution (ATS) alone (without any drugs or polymers), whereas the experimental groups received topical instillation with a single dose of 50 µL of ophthalmic solutions containing CsA, p(MPC), p(MPC_1_-*co*-DMA_1_), p(MPC_3_-*co*-DMA_1_), and p(MPC_6_-*co*-DMA_1_). A Pre group (healthy eyes) and a DED group (as-induced dry eyes) were also observed for comparison with Ctrl group and test groups.

The therapeutic efficacy of p(MPC) and of different p(MPC-*co*-DMA) formulations was determined based on ophthalmic evaluations that were performed before and after topical administration. The bilateral eyes of 36 rabbits were then measured at predetermined time intervals during the following 4 days. Corneal fluorescein staining studies were used for clinical observations. The stained ocular surface was examined and graded using slit-lamp biomicroscopy (Topcon Optical, Tokyo, Japan) after topical application of 1% fluorescein sodium into the conjunctival sac. The intensity of fluorescence was then measured under cobalt blue light for slit-lamp analysis. The amount of fluorescence staining in the cornea was measured for three random areas on each rabbit eye using a standard four-point scale (0 = none, 1 = mild, 2 = moderate, and 3 = severe). The staining was measured in grades (*n* = 6) from 0 to 9.

Details of other experimental procedures, including the synthesis of DMA, 3-(4,5-dimethylthiazol-2-yl)-5-(3-carboxymethoxyphenyl)-2-(4-sulfophenyl)-2 H-tetrazolium (MTS) test, live/dead assay, comet assay, corneal topographer assay, corneal endothelial cell density measurement, hematoxylin and eosin (H&E) study, terminal deoxynucleotidyl transferase dUTP nick end labeling (TUNEL) assay, DCFH-DA immunofluorescence staining, IL-6 and TNF-α immunofluorescence staining, Integrated Clinical Platform (I.C.P.) ocular surface analysis and Schirmer tear test are provided in Supporting Information.

### Statistical analysis

All results are reported as mean ± standard deviation (SD). The significance is measured using a one-way analysis of variance (*p* < 0.05).

## Results and discussion

### Characterization studies

**Fig. 1:**
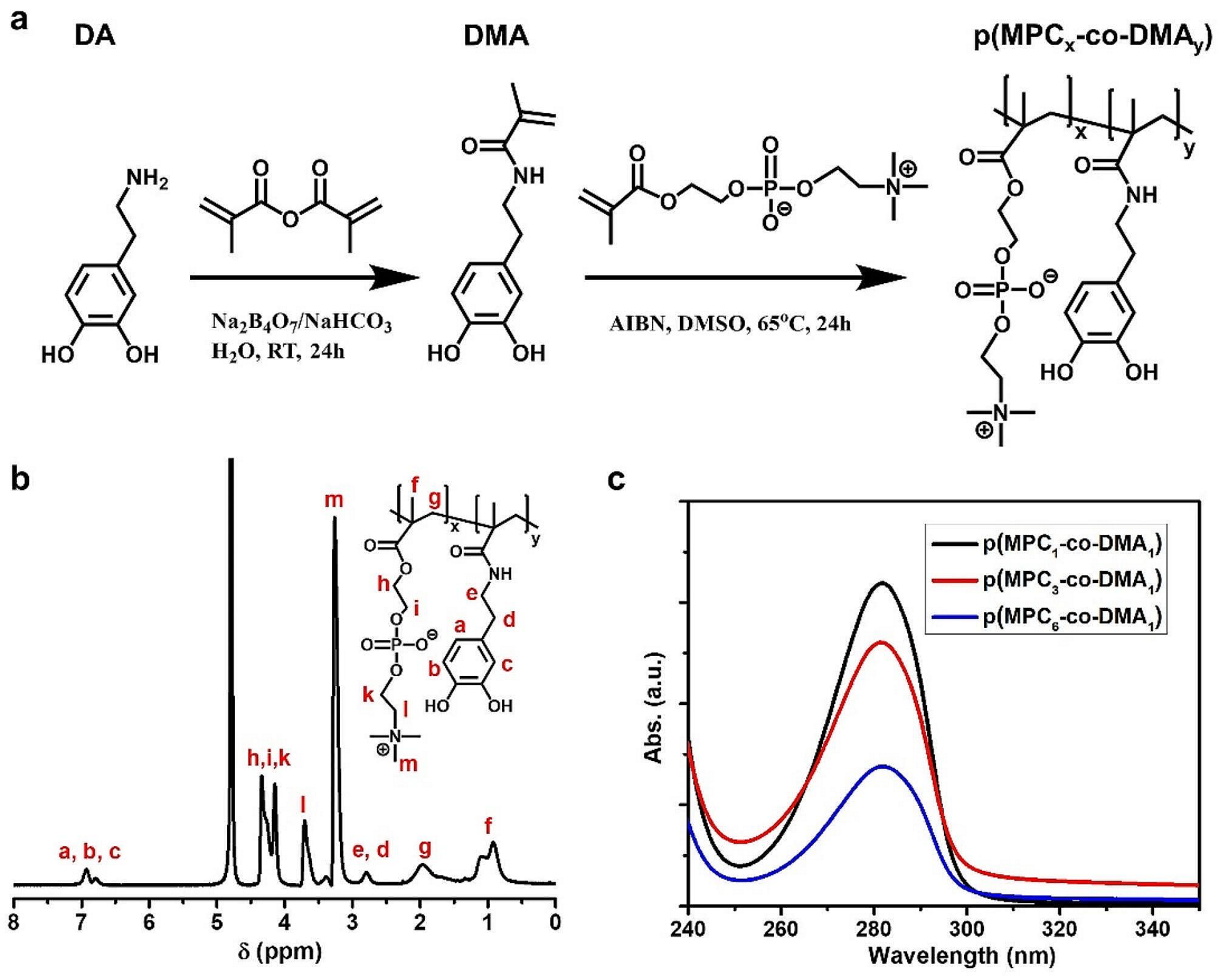
Synthesis routes and characterizations of copolymers. (**a**) The preparation of DMA monomer and p(MPC-*co*-DMA) copolymers. (**b**) ^1^H NMR spectrum of p(MPC_1_-*co*-DMA_1_) in D_2_O. (**c**) UV-VIS spectra of p(MPC-*co*-DMA) (1 wt%) with different compositions in PBS at pH 7.4

p(MPC-*co*-DMA) was synthesized and characterized as shown in Fig. 1a. The successful synthesis of DMA monomer was confirmed as shown in Figure S1. FTIR spectra of DMA monomer depicted the characteristic peak at 1650 cm^− 1^, which signifies the stretching vibration of C = O in amide group (Figure S2). The result suggested the amidation reaction and polymerizable unit had been conjugated with catechol motif. In addition, the peak at 3225 cm^− 1^ was assigned to -OH stretching vibration of catechol unit. The result indicates the availability of DMA for adhesive function. Prior to copolymerization, given that MPC monomer has low solubility in DMSO solvent, the successful p(MPC) synthesis was confirmed using NMR spectra analysis. As shown in Figure S3, the NMR spectrum of p(MPC) showed no proton signals of residual vinylic double bond at δ = 5.5 − 6.5 ppm, indicating that the resultant powder from our synthesis was in polymer configuration.

A series of copolymers with varying compositions were produced by using the feeding molar ratios of MPC and DMA of 1:1, 3:1, and 6:1. Figure 1b and Figure S4 display the ^1^H NMR spectra of various p(MPC-*co*-DMA) samples using D_2_O as the solvent. The signals at 6.79 and 6.93 ppm correspond to 3,4,6-trihydrophenyl groups of DMA. In addition, the peaks at 3.26, 3.71, 4.14 and 4.34 ppm are attributed to methylene groups and the methyl groups in MPC. The functional groups of all polymer compositions were further analyzed using FTIR spectra. Figure S5 showed the characteristic bands at 1244, 1170 and 1082 cm^− 1^, which are attributed to phosphate groups in MPC moiety. Additionally, the absorbance peak at 967 cm^− 1^ is assigned to the antisymmetric stretches of the C − N bonds in the N-(CH_3_)_3_ of MPC moiety. Notably, in various p(MPC-*co*-DMA) polymers, a novel peak at 1527 cm^− 1^ may represent the stretching vibration of C-N bond in amide motif of DMA. To this end, the NMR and FTIR spectra have confirmed the successful synthesis of the p(MPC-*co*-DMA) via free-radical copolymerization. For all of the polymer samples, the actual ratios of MPC to DMA are determined by dividing the integrated area of the aromatic protons of DMA (δ = 6.79 to 6.93 ppm) by the integrated area of the characteristic peaks for MPC (δ = 3.26 ppm). For feeding ratios of MPC to DMA of 1:1, 3:1, and 6:1, the respective ratios for the resultant samples is 2.77:1, 4.49:1 and 7.09:1 (Table S1). Accordingly, the highest DMA content is achieved at a value of 26.5 mol% for p(MPC_1_-*co*-DMA_1_). In nature, enriched catechol residues have been documented with values of 30 mol% in the interfacial mussel’s foot proteins (mfp-3 and mfp-5) [41]. Therefore, p(MPC_1_-*co*-DMA_1_) closely resembles a mussel’s foot protein in term of catechol content. The respective catechol content of p(MPC_3_-*co*-DMA_1_) and p(MPC_6_-*co*-DMA_1_) is 18.2 and 12.4 mol%. Figure 1c further confirms the higher catechol content in the copolymers at 1 wt% in the order of molar ratio 6:1 < 3:1 < 1:1, which is consistent with the molar ratio recorded using ^1^H NMR. In addition, the catechol content of copolymers quantified by UV-VIS (Fig. 1c, Figure S6 and Table S1) can support the data of NMR spectra (Fig. 1b and Table S1).

The number-average molecular weight (*M*_n_) of the copolymers determined by GPC is shown in Figure S7 and Table S1. The length of the polymer chain decreases as MPC feeding mass increases, as shown in Experimental section. It is difficult to dissolve DMA homopolymers in organic solvents, and the polymers only demonstrate limited solubility in dimethylformamide [42]. Without sufficient protection, radical scavenging ability by catechol hinders the polymerization reaction because radicals are quenched by semi-quinone radicals, leading to the formation of intra- or inter-crosslinked structure [43, 44]. Therefore, the copolymers with greater MPC feeding content may easily form a long polymer chain. Zhang and colleagues also found that the solubility of copolymer at the molar ratio of 1:3 (ratio between MPC: DMA) was limited at 4 mg/mL [38]. In this study, our attempt to increase DMA content with feeding ratios 1:3 and 1:6 showed a sight of catechol oxidation (a darken color in the polymerization solution) as shown in Figure S8a. Additionally, the vinylic signals of DMA at δ = 5–6 ppm were still visible after the reaction, indicating the low polymerization efficacy of MPC: DMA feeding ratios at 1:3 and 1:6 (Figure S8b). Therefore, the highest DMA portion was only observed at 1:1 for this study.

### Friction measurements

**Fig. 2:**
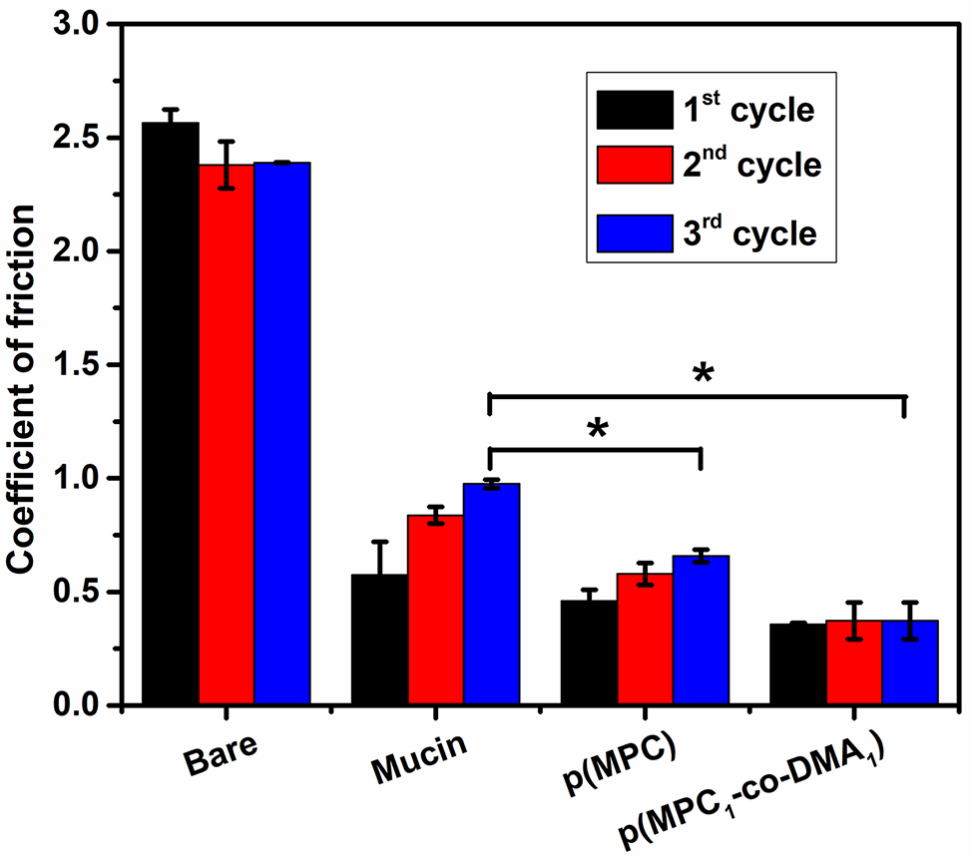
Hydration lubrication evaluation of as-prepared polymers. COF of silicon tube (Bare), Mucin-coated, p(MPC)-coated and p(MPC_1_-*co*-DMA_1_)-coated silicon tubes in DI water. The friction tests were performed in each sample at a normal load of 2 N and a sliding speed at 150 mm/mL after three sliding cycles. Values are mean ± SD (*n* = 3). *p < 0.05 vs. mucin

Lubricating agents can act as an osmoprotectant to ease dry eye discomfort by restoring the physiological osmolarity of tear film and decreasing the effect of hyperosmotic distress to corneal cells [45]. Therefore, the lubrication properties of mucin and synthesized polymers on silicon tubes were investigated. For the pristine silicon tube (Bare), the COF was constant at ∼ 2.5 for all three sliding cycles (Fig. 2). After mucin deposition, the COF value for the surface initially decreases to ∼ 0.57, and then gradually increases to ∼ 0.98 after the 2nd and 3rd cycles. The increase in COF tendency was also observed in other polymer coating substrates. Because a mucin layer was generated on coating surface via non-specific adsorption, the interfacial shear force between the coating and the silicon sheet could remove a portion of adsorbed mucin macromolecules after each sliding cycle. As a result, the stability of the lubrication that the mucin-bound surface provides is compromised. For a polymer-bound surface, the smoothness of the surfaces in which p(MPC) is deposited surfaces increases to give a COF of 0.46 to 0.66 after three cycles but surfaces on which p(MPC_1_-*co*-DMA_1_) is deposited experience only a slight change in COF from 0.36 to 0.37. The difference in COF values for the two polymer compositions probably reflects the functionality of DMA. Catechol has a high adhesive strength on a variety of surfaces [41], which could increase the stability of the coating against shear stress. A significantly lower COF value for p(MPC)- and p(MPC_1_-*co*-DMA_1_)-coated surfaces than for mucin-bound surfaces at the final sliding cycle (*p* < 0.05) indicates the enhanced lubrication properties of zwitterionic polymers. Considering the high dipole moment of zwitterionic pendants, the strong ionic-driven hydration shell surrounding quaternary amine N^+^(CH_3_)_3_ and phosphonate PO_4_^−^ is not easily squeeze out under shear or compression, facilitating a greater degree of viscous dissipation than for “non-hydration” water [46, 47]. Therefore, the interfacial friction for surfaces on which MPC is deposited is lower than the friction for mucin-bound surfaces.

### Mucoadhesive studies

Commercial porcine stomach mucin was used to study the mucoadhesive properties of p(MPC-*co*-DMA) copolymers. The interaction between catechol-containing copolymer and mucin was first measured using UV-VIS spectroscopy. 100 µL of copolymer solutions (10 mg/mL) were added to 1 mL of mucin solutions (1 mg/mL) to achieve a mass ratio of 1:1 in PBS buffer at pH 7.4. As shown in Figure S9a, the mucin solution exhibits a strong absorbance signal at 260 nm. There is no significant difference in the position of the absorbance peak for mucin and mucin-p(MPC). In contrast, UV-VIS demonstrated a slight red shift if mucin solution is mixed with catechol-functionalized polymer. The absorbance maxima for mucin shifts toward a lower wavelength region and there is an increase in the catechol content, which demonstrates that a mucin-catechol complex forms [34, 48, 49]. This behavior is also noted by other study, suggesting a change in peptide strands of mucin molecules and their hydrophobicity [48]. As a zwitterionic moiety, MPC segments of the copolymer resist non-specific interaction with biomacromolecules [50]. Hence, mucin-copolymer complex formation is solely contributed by DMA segments. As the catechol degree increases, more reactive sites are available for conjugation. Furthermore, copolymers separately recorded show no sign of catechol oxidation [51, 52], displaying only one absorbance peak centered at 280 nm in PBS buffer (Figure S9a). Hence, bidentate catecholic hydroxyls can induce strong hydrogen bonds [30, 49], allowing initial contact for mucin complexation.

Mucins play a vital role in the innate protection of the eyes [53]. Hence, the mucoadhesive properties of p(MPC-*co*-DMA) were further studied using a mucin-deposited substrate prior to in vitro and in vivo studies. Mucins were coated onto a plasma-treated silicon wafer by passive adsorption for 1 h. To verify the stability of the coating, WCA measurement was used to qualify the relative hydrophilicity after mucin deposition. Figure S9b shows that there is increased wetting of the surface of the silicon wafer so there is a significant reduction in the value of the WCA from 68° to 26.4°. Mucins exhibit a high binding affinity with water molecules [54]. This renders coated silicon surface with improved hydrophilicity. After deposition with mucin, copolymers were allowed to deposit on the substrates for 1 h. Interestingly, p(MPC_1_-*co*-DMA_1_) further induced wetting of mucin film (WCA at 23.2°), while a similar result was not observed in samples coated with other polymer compositions. Because zwitterions can exhibit superior wetting via ionic solvation [15], the decrease in the WCA implies the abundance of MPC segments on the mucin layer of p(MPC_1_-*co*-DMA_1_).

**Fig. 3:**
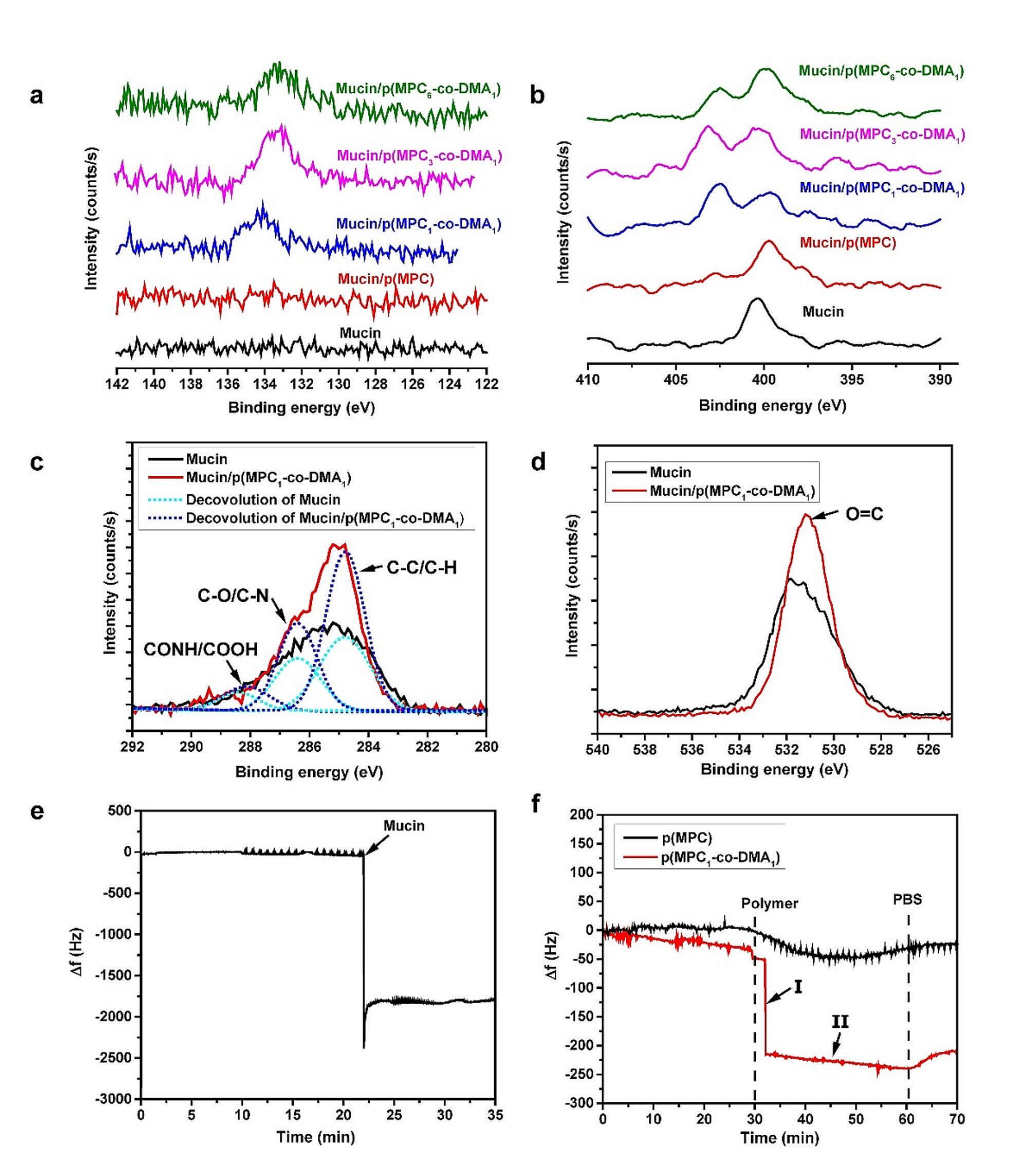
Mucoadhesion evaluation of as-prepared polymers. XPS spectra of the modified substrates: Core-level binding energy of elements: P2p (**a**), N1s (**b**), C1s (**c**), and O1s (**d**). Changes in frequency Δf with time were recorded with eQCM for mucin deposition (**e**) and the interaction between polymers on mucin layer (**f**)

The surface elemental composition of the modified surface was investigated using XPS (Fig. 3). The characteristic phosphorus P2p signal at 133.8 eV is only attributed to the phosphate group of MPC [55]. Figure 3a shows that the signal is absent for the mucin layer. For different polymer compositions, only catechol-containing copolymers are immobilized on mucin-coated surfaces. The high-resolution spectra for the phosphorus to carbon ratio (P/C) also shows that there is an increase from 0.07 to 0.14 (Table S2), with increasing degree of catechol: p(MPC) < p(MPC_6_-*co*-DMA_1_) < p(MPC_3_-*co*-DMA_1_) < p(MPC_1_-*co*-DMA_1_). The N1s core-level spectra features a peak for mucin at 400.3 eV, which is associated with the primary and secondary amines at the N-terminus and peptide bonds in mucins (Fig. 3b) [53, 54]. After the copolymer deposition, a new N1s peak at 402.7 eV appears, which demonstrates the existence of the quaternary amine N^+^(CH_3_)_3_ in MPC. The presence of phosphorus and charged N^+^ are clear evidence that zwitterionic moieties are immobilized [58]. Moreover, introducing acrylamide CH-NH-CH section into DMA increases N elemental signals on mucin-deposited surface. As a result, the atomic ratio of nitrogen to carbon (N/C) is greater for samples on which a copolymer is deposited than for the mucin layer. These results demonstrate that DMA features an anchoring functionality.

.

Catechols can react with thiol and amine via Schiff base and Michael addition reactions [59]. Thiol-containing residues of mucins have been reported by other studies [9, 60]. However, the cysteine components of mucin vary between purification batches due to the variations in animals or purification efficiency [61]. Furthermore, cysteine is also not as abundant as amine residues [34]. Thus, the mucoadhesive properties of polymers are assumed to be most obvious between catechol and amine portions in mucins. To investigate this possibility, the core-level spectra of C1s and O1s were further analyzed in the mucin layer with and without deposition of p(MPC_1_-*co*-DMA_1_) (Fig. 3c and d). The C1s spectral signal for mucin layer shows three respective peak components for C-C/C-H, C-O/C-N, and CONH/COOH at 284.8, 286.4, and 288.2 eV (Fig. 3c) [56, 62]. In the p(MPC_1_-*co*-DMA_1_) spectrum, the peak intensities for C-C/C-H and C-O/C-N increase, which indicates the presence of the copolymer backbone and the tethered residues (acrylamide in DMA and acrylate in MPC) on mucin. In the O1s spectra, there is an increase in O = C for a binding energy of 531.2 eV in p(MPC_1_-*co*-DMA_1_) [63, 64], which shows that catechol moieties were oxidized (Fig. 3d). Interestingly, catechol moieties were in a reduced state as shown in UV-VIS spectra, which differs from the result observed in XPS. The oxygen-induced conversion of catechol to quinone can occur above pH 5; however, the process is rather dull, requiring days of monitoring [57]. Hence, it is likely that catechol-to-quinone conversion did not occur after hours under a physiological condition during UV-VIS measurement. Furthermore, amine-rich mucins may accelerate the oxidation of catechol residues by promoting Michael addition and Schiff base reactions, similar to the co-deposition between DA and polyethyleneimine in the work of Xu and colleagues [62].

Real-time measurement of p(MPC_1_-*co*-DMA_1_) adsorption on the mucin adlayer used eQCM to analyze adhesion behavior. The reduction in frequency shift (Δf) is associated with adsorbed mass on the sensor. Herein, we first examined the adlayer formation of mucin used in this study. Mucin (0.025 mg/mL) was injected and allowed to adsorb in the flow cell for 35 min, forming a mucin adlayer with a stable mass density, as shown in Fig. 3e. The adlayer was then rinsed with PBS for 30 min to remove loosely-bound mucin. The polymer compositions were then allowed to flow for 30 min before rinsing with PBS as shown in Fig. 3f. For p(MPC) homopolymer, there was a gradual decrease in Δf after injection, then a small increase and halt at a mass density of 21.6 ng/cm^2^. The main driving force for non-specific adsorption of p(MPC) is Van der Waal forces, which are common but relatively weak secondary bonds in close proximity [65]. After p(MPC_1_-*co*-DMA_1_), there was an abrupt decrease (phase I) followed by a steady decrease (phase II) in Δf. In phase I, the copolymer passively diffuses and is absorbs onto mucin adlayer, and the interaction between catechol and mucin provides anchoring sites to allow stable adhesion of the copolymer. In Phase II, the catechol groups that remain on the adsorbed copolymers also allow inter-chain interactions with free-floating chains, leading to an increase in the adsorbed mass density. As a result, the final mass load for the copolymer is 213.2 ng/cm^2^, which is approximately 10 times the value for p(MPC). To sum up, the adhesive mechanisms for catechol-functionalized polymers have three aspects: (1) catecholic functionalization promotes the immobilization of zwitterions on the mucin surface, as shown by the XPS spectra; (2) bidentate catecholic residues of the copolymer allow strong and instantaneous hydrogen bonding with the mucin layer; and (3) the catechol-amine adducts that gradually form between the mucin and the copolymer increase the mucoadhesive properties of the copolymers.

### In vitro biocompatibility tests

Prior to the antioxidant and anti-inflammatory evaluation, the cytotoxicity of p(MPC) and p(MPC-*co*-DMA) is determined. The biocompatibility of all synthetic polymers is determined using a MTS assay and live/dead staining. For this work, SIRC cells from the cornea of normal rabbit was used. The mitochondrial dehydrogenase activity (MTS activity) for the Ctrl group was defined as 100%. The metabolic activities and viability levels of SIRC cells after exposure to polymer samples were depicted in Figure S10 and S11. After two days of incubation, all the polymer samples did not alter the cell morphology regardless of their concentration (ranging from 0.1 to 1 mg/mL) (Figure S10a and b). Quantitative data for MTS activity and live/dead assay also supported data from the microscopic images in that the values for metabolic activity and cell viability are 95% of the values for the Ctrl group (Figure S10 and S11). In addition to cell cytotoxicity, an alkaline comet assay was conducted to investigate the potential genotoxicity of the polymers. Figure S12 indicated intact nuclei with smooth edges in all samples. Moreover, the tail lengths of comet in all compositions showed no apparent difference with the Ctrl (0.9 ∼ 1.2 μm). The result shows that no DNA breakage was caused by the polymers [66, 67]. Overall, the synthetic polymers used in this study exhibited no harmful effect on SIRC cells. Therefore, the in vitro and in vivo studies were conducted and evaluated using the highest polymer concentration of 1 mg/mL.

### Antioxidant and anti-inflammatory activity studies

**Fig. 4:**
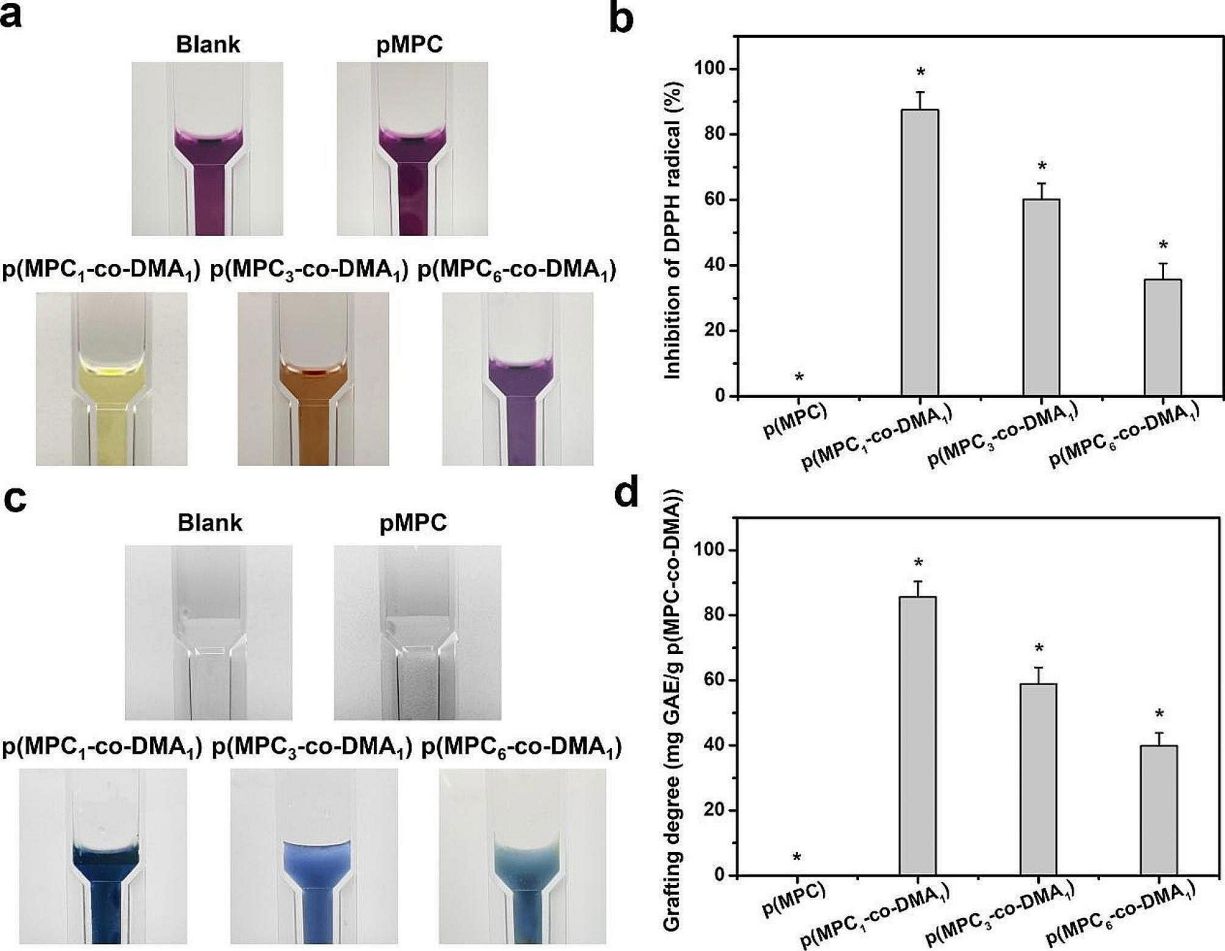
Anti-oxidation evaluation of as-prepared polymers. Photographs of the reaction of DPPH reagent (**a**) and Folin-Ciocalteu reagent (**c**) with the test samples including p(MPC), p(MPC_1_-*co*-DMA_1_), p(MPC_3_-*co*-DMA_1_) and p(MPC_6_-*co*-DMA_1_). DPPH scavenging activity (**b**) and total phenolics content (**d**) of the various samples were analyzed by UV-VIS spectrophotometry. Values are mean ± SD (*n* = 5). *p < 0.05 vs. all groups. The blank group: without p(MPC-*co*-DMA) sample

An increase in the amount of ROS is a major factor of the DED etiology [68]. The over-expression of intracellular ROS on the ocular surface has various adverse outcomes, including DNA, lipids, protein damage and cell apoptosis [69]. To determine the antioxidant activity of the polymers, this study uses DPPH to determine the free radical scavenging ability for various p(MPC-*co*-DMA) groups. The results are shown in Fig. 4a and b. DPPH antioxidant solution has a maximum absorbance at 517 nm [70]. As the radical scavenging capability of antioxidant agent increases, the color of DPPH solution gradually changed from dark purple to light yellow [71]. Quantitatively, the respective radical inhibition percentages of p(MPC_1_-*co*-DMA_1_), p(MPC_3_-*co*-DMA_1_), and p(MPC_6_-*co*-DMA_1_) was 87.52 ± 5.36%, 60.14 ± 4.89% and 35.63 ± 4.94%. In contrast, the value of p(MPC) was similar to blank sample. These results highlighted that the antioxidant capability was closely associated with the degree of catechol groups within copolymers, which has obtained wide recognition in other literature [38, 44]. To support this assumption, we continued to conduct Folin-Ciocalteu assay to determine the polyphenol content of p(MPC-*co*-DMA). The color of a p(MPC_1_-*co*-DMA_1_) solution turned darker blue as shown in Fig. 4c, implying a higher level of polyphenols [72]. The polyphenol content at 85.7 ± 4.7 mg, 59.0 ± 5.0 mg, and 39.9 ± 3.9 mg for gallic acid equivalent (GAE)/g polymer decreases in the order (Fig. 4d): p(MPC_1_-*co*-DMA_1_) > p(MPC_3_-*co*-DMA_1_) > p(MPC_6_-*co*-DMA_1_).

**Fig. 5:**
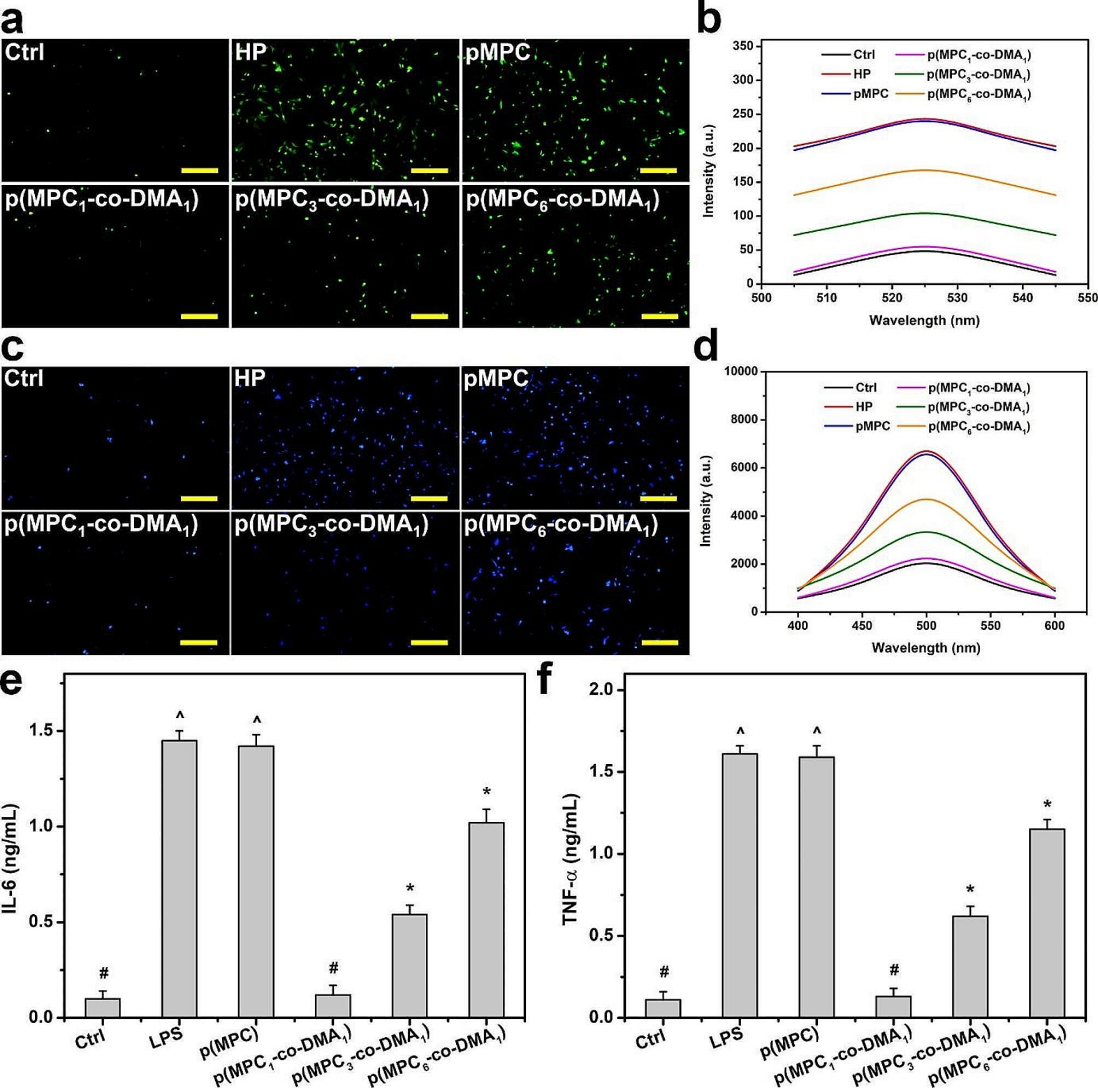
In vitro anti-oxidation and anti-inflammation of as-prepared polymers. (**a**) Representative fluorescent images and (**b**) intracellular levels of ROS measured by the fluorescence intensity of DCFH-DA of the SIRC cells after incubation with different samples for 24 h and further exposure to H_2_O_2_ for 24 h. (**c**) Representative fluorescent images of the SIRC cells and (**d**) intracellular levels of calcium measured by the fluorescence intensity of Fura-2, AM after incubation with different samples for 24 h and further exposure to H_2_O_2_ for 24 h. Levels of (**e**) IL-6 and (**f**) TNF-α from the p(MPC-*co*-DMA) samples (p(MPC), p(MPC_1_-*co*-DMA_1_), p(MPC_3_-*co*-DMA_1_) and p(MPC_6_-*co*-DMA_1_)). Scale bars: 100 μm. Values are mean ± SD (*n* = 5). *p < 0.05 vs. all groups; #*p* < 0.05 vs. LPS, p(MPC), p(MPC_3_-*co*-DMA_1_) and p(MPC_6_-*co*-DMA_1_) groups; ^*p* < 0.05 vs. Ctrl, p(MPC_1_-*co*-DMA_1_), p(MPC_3_-*co*-DMA_1_) and p(MPC_6_-*co*-DMA_1_) groups

Cellular models of oxidative stress were adopted to assess the antioxidant capabilities of p(MPC-*co*-DMA) materials in vitro. DCFH-DA, which is a cell-permeable non-fluorescent probe, can be hydrolyzed to become non-permeable DCFH within cells. DCFH can be further oxidized by increased intracellular ROS (superoxide O_2_^−^), forming visible fluorescent signals as shown for HP group (Fig. 5a). The fluorescent signals from cells decrease significantly after two days of incubation with catechol-containing polymers. The greater the concentration of catechols in p(MPC_1_-*co*-DMA_1_), the more the ROS level reduces to the value for the Ctrl group (Fig. 5b). Catechol can quench free radicals by supplying hydrogen atoms from the phenolic hydroxyl residues, but catechol can also reduce other reactive agents through electron transfer, and the resulting phenoxyl radical can further interact with the second radical to form stable quinone structures [73]. The regulation of calcium has a crucial role in chemical signaling, thus the over-expression of intracellular calcium is considered a contributing factor leading to cell death [74, 75]. Therefore, further studies on intracellular calcium levels were conducted to verify cell survivability (Fig. 5c and d). The intracellular calcium level after H_2_O_2_ treatment decreased significantly when cells were co-incubated with p(MPC-*co*-DMA) copolymers in the order: Ctrl < p(MPC_1_-*co*-DMA_1_) < p(MPC_3_-*co*-DMA_1_) < p(MPC_6_-*co*-DMA_1_) < p(MPC) = HP. These results confirm that catechol-functionalized polymers inhibit ROS damage.

Inflammation is an underlying factor for DED [69]. Hence, the pro-inflammatory factors (IL-6 and TNF-α) were further examined. Lipopolysaccharide-induced cells were also used as a disease control group (LPS) to mimic DED symptoms. As depicted in Fig. 5e and f, the expression of IL-6 and TNF-α decreases significantly (*p* < 0.05) for LPS groups that are subject to p(MPC-*co*-DMA) treatment. Moreover, p(MPC_1_-*co*-DMA_1_)-treated group exhibits the highest anti-inflammatory capability of the catechol-containing groups, which demonstrates their antioxidant capacity.

### Corneal retention studies

In this study, the normal rabbit corneas were monitored under a slit lamp for h12 and 4 days post-instillation to validate any corneal injury caused by polymers. The rabbit eyes receiving topically instilled polymeric drops showed no signs of corneal redness, edema, or inflammation (Figure S13). Quantitatively, all observations of the modified Draize test resulted in scores of zero as shown in Table S3. Furthermore, intraocular pressure (IOP) value after polymer instillment remained comparable with Ctrl group, which confirmed the safety of the synthetic polymers (Figure S14).

**Fig. 6:**
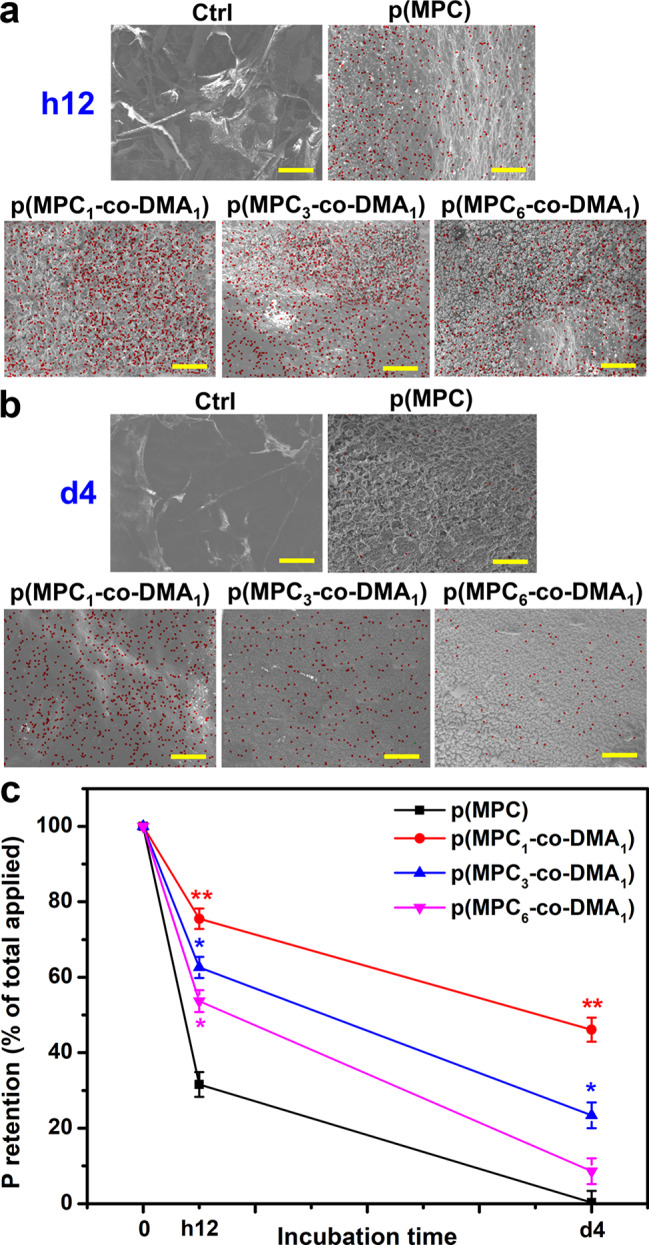
In vivo mucoadhesion assessment of nanoformulations. SEM images of healthy eyes at (**a**) h12 and (**b**) 4 days post-instillation of different formulations. EDS mapping of phosphorus distribution for the corresponding healthy eyes. Scale bars: 10 μm. (**c**) Corneal retention of P was analyzed by ICP-OES with the various p(MPC-*co*-DMA) samples. Values are mean ± SD (*n* = 6). *p < 0.05 vs. p(MPC) group; **p < 0.005 vs. p(MPC) group

The bio-adhesive properties of p(MPC-*co*-DMA) copolymers were further evaluated in vivo. In this investigation, the content of p(MPC-*co*-DMA) retained on the cornea was examined by SEM-EDS after h12 and 4 days of topical administration of nanotherapeutic formulations (Fig. 6a). No elemental phosphorous (red dot) was detected in the Ctrl group. The meibomian lipid layer is constituted by the structural organization of polar lipids (of which phospholipids are the most important) [76]. However, the precorneal tear film is not a part from corneal tissue. This result may explain the absence of phosphorus signal on ocular surface. Hence, the measurement for elemental phosphorous can only be attributed to phosphorylcholine pendants from MPC-containing polymers. For p(MPC) group, the phosphorus portion was ∼ 31% after h12 of treatment. In contrast, at least 50% of the total phosphorus signal, which corresponds to p(MPC-*co*-DMA), remained visible on the ocular surface (75%, 62%, and 53% for the feeding ratios of 1:1, 3:1 and 6:1, respectively). The results suggested the concentration of catechol significantly affect retention efficiency. Remarkably, phosphorus coverage of p(MPC_1_-*co*-DMA_1_) remained at about 46%, but the phosphorus coverage of p(MPC_3_-*co*-DMA_1_) and p(MPC_6_-*co*-DMA_1_) decreased significantly (23% and 8%, respectively), as shown in Fig. 6b. As demonstrated in the mucin model, the initial attachment of the copolymers was due to the presence of bidentate hydroxyls in reduced catechol. However, covalent solidification of amine-catechol adducts is essential for long-lasting mucoadhesion [34]. The abundant catechol residues within p(MPC_1_-*co*-DMA_1_) probaly promote an inter-chain interaction via phenol-phenol coupling [33], leading to heightened P retention percentage.

### Therapeutic efficacy studies

**Fig. 7:**
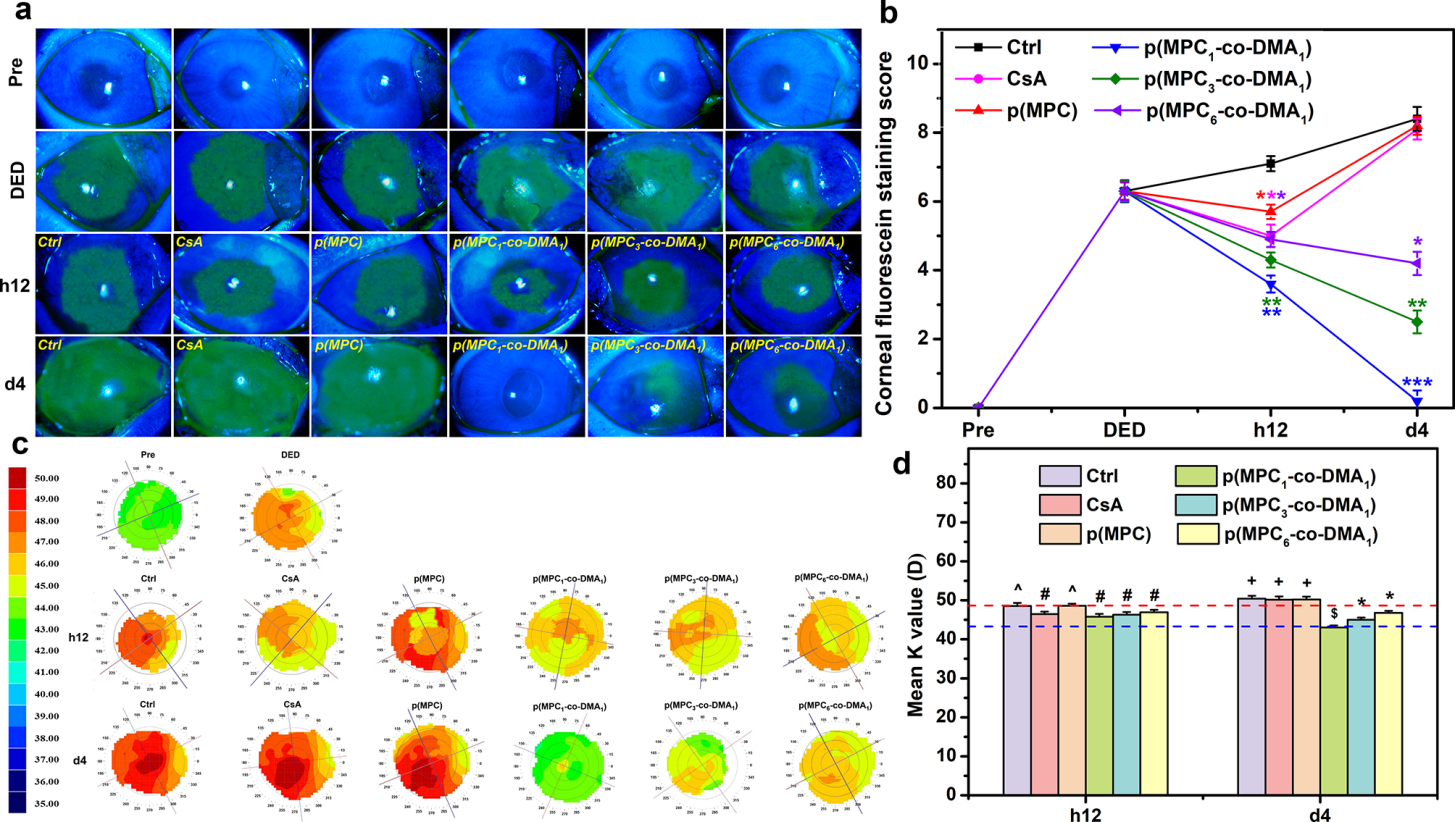
In vivo therapeutic effects of nanoformulations. (**a**) Corneal fluorescein staining images and (**b**) fluorescent staining scores of rabbit eyes at preoperation (Pre), after dry eye (DED) induction, and those with experimentally induced DED after topical administration of different formulations for h12 and 4 days. Dry eye animals receiving ATS without polymer and drug were control groups (Ctrl). *p < 0.05 vs. Ctrl group; **p < 0.005 vs. Ctrl group; ***p < 0.001 vs. Ctrl group. (**c**) Corneal topographic values and (**d**) mean K value from corneal topography of rabbit eyes after h12 and 4 days of topical instillation of different formulations. The blue and red dash lines represent the Pre and DED group values, respectively. Values are mean ± SD (*n* = 6). *p < 0.05 vs. all groups; +*p* < 0.05 vs. Pre, DED, CsA, p(MPC_1_-*co*-DMA_1_), p(MPC_3_-*co*-DMA_1_), and p(MPC_6_-*co*-DMA_1_) groups; #*p* < 0.05 vs. Pre, Ctrl, and p(MPC) groups; ^*p* < 0.05 vs. Pre, DED, p(MPC_1_-*co*-DMA_1_), p(MPC_3_-*co*-DMA_1_), and p(MPC_6_-*co*-DMA_1_) groups; $*p* < 0.05 vs. Pre, Ctrl, CsA, p(MPC), p(MPC_3_-*co*-DMA_1_), and p(MPC_6_-*co*-DMA_1_) groups

The therapeutic effect of the synthetic polymer was investigated in BAC-induced DED model. BAC, which is a preservative used in many ophthalmic topical solutions, can accelerate tear film breakage and evaporation [77]. Rabbits with dry eyes exhibited corneal/conjunctival injury and insufficient tear secretion at the end of the induction phase, similar to those of DED patients. An examination of the ocular surface and tear film in a clinical ophthalmology exam can indicate the progression of dry eye diseases [78]. Clinical criteria for DED, including a fluorescein vital staining score of ≥ 3 and a Schirmer test value of ≤ 5 mm, were used to validate the recovery of rabbit eyes after treatment using different polymer compositions. For the untreated group (Ctrl group), the DED rabbits that receiving ATS alone exhibited the greatest fluorescent intensity for the experiment. An immunomodulatory agent (CsA) was used as a reference for comparison with the polymers. CsA can alleviate the symptoms of dry eye and the underlying inflammatory pathologic state [77]. However, CsA possesses low solubility and bioavailability [79], limiting its potential therapeutic effect for DED treatment. The CsA group only showed a reduction in the area of corneal fluorescein staining after h12 of administration (Fig. 7a). The short-term therapeutic action was also observed in homopolymer p(MPC). As shown in Fig. 7b, p(MPC) exhibited a reduction (*p* < 0.05) in fluorescein staining score after h12 of administration. The instillation of hydrophilic p(MPC) can replenish moisture on the ocular surface and stabilize the osmolarity of tear film [25], but did not adhere well and did not produce an anti-oxidative action to inhibit the progression of DED. Hence, without sufficient bioavailability and bio-adhesive mechanism, CsA and p(MPC) groups have similar scores to those for the untreated DED model (Ctrl) at 4 days postoperatively. In contrast, the positive fluorescent signals for p(MPC_1_-*co*-DMA_1_), p(MPC_3_-*co*-DMA_1_), and p(MPC_6_-*co*-DMA_1_) decreased linearly after h12 and 4 days postoperatively, signifying a gradual mitigation of corneal epithelial damage. Furthermore, there was a correlation between catechol concentration and the therapeutic effect of p(MPC-*co*-DMA). With the highest synergistic antioxidant/anti-inflammatory effect demonstrated in vitro, the fluorescein vital staining score in p(MPC_1_-*co*-DMA_1_)-treated groups reduced to ∼ 0 (*p* < 0.001), suggesting the most pronounced therapeutic effect.

In ophthalmology, corneal imaging is used to detect and treat various ocular illnesses. In addition, corneal topography can be used to monitor changes in response to ocular disease progression. As shown in Fig. 7c and d, the green signal in Pre group is almost equally distributed, indicating a minimal corneal abnormality. On the contrary, DED and Ctrl groups displayed a heterogeneous distribution of green, yellow, orange, and bright red signals due to inflammation, suggesting a horizontal wavefront aberration. A comparison of the results for all the groups shows that the corneal topological map and curvature values for the p(MPC_1_-*co*-DMA_1_) group most resemble those for the Pre group and demonstrate the greatest therapeutic effect for DED.

DED-associated inflammation can accelerate corneal endothelial cell loss and change cellular morphology [80]. Hence, specular microscopic images and the endothelial cell density of rabbit eyes in response to topical instillation of the polymers were investigated at h12 and 4 days postoperatively. In the Pre group, healthy endothelial cells on Descemet’s membrane displayed a characteristic hexagonal shape and were closely packed together (Figure S15a). On the contrary, morphological abnormality of endothelial cells was detected in the DED model. The change in cellular morphology became more evident in Ctrl and p(MPC) groups, along with a significant reduction (*p* < 0.05) in endothelial cell density compared with DED group (Figure S15a and b). For other groups, because CsA and p(MPC-*co*-DMA) nanoformulations are bioactive (antioxidant/anti-inflammation), the exposure of DED-induced eyes to the polymers can inhibit the continuous deterioration of endothelial cell density after h12 of instillation. However, insufficient doses and the lack of retention mechanisms may hamper the bioavailability of CsA [12, 13], which limits its long-term therapeutic effect as depicted in Figure S15b. p(MPC-*co*-DMA) copolymers with their superior mucoadhesion and antioxidation can further prevent corneal endothelial damage at 4 days postoperatively. Our findings indicated that among all experimental groups, p(MPC_1_-*co*-DMA_1_) not only therapeutically promoted epithelial regeneration, but also efficiently preserved cell morphology and density of corneal endothelium during 4 days of clinical observation.

**Fig. 8:**
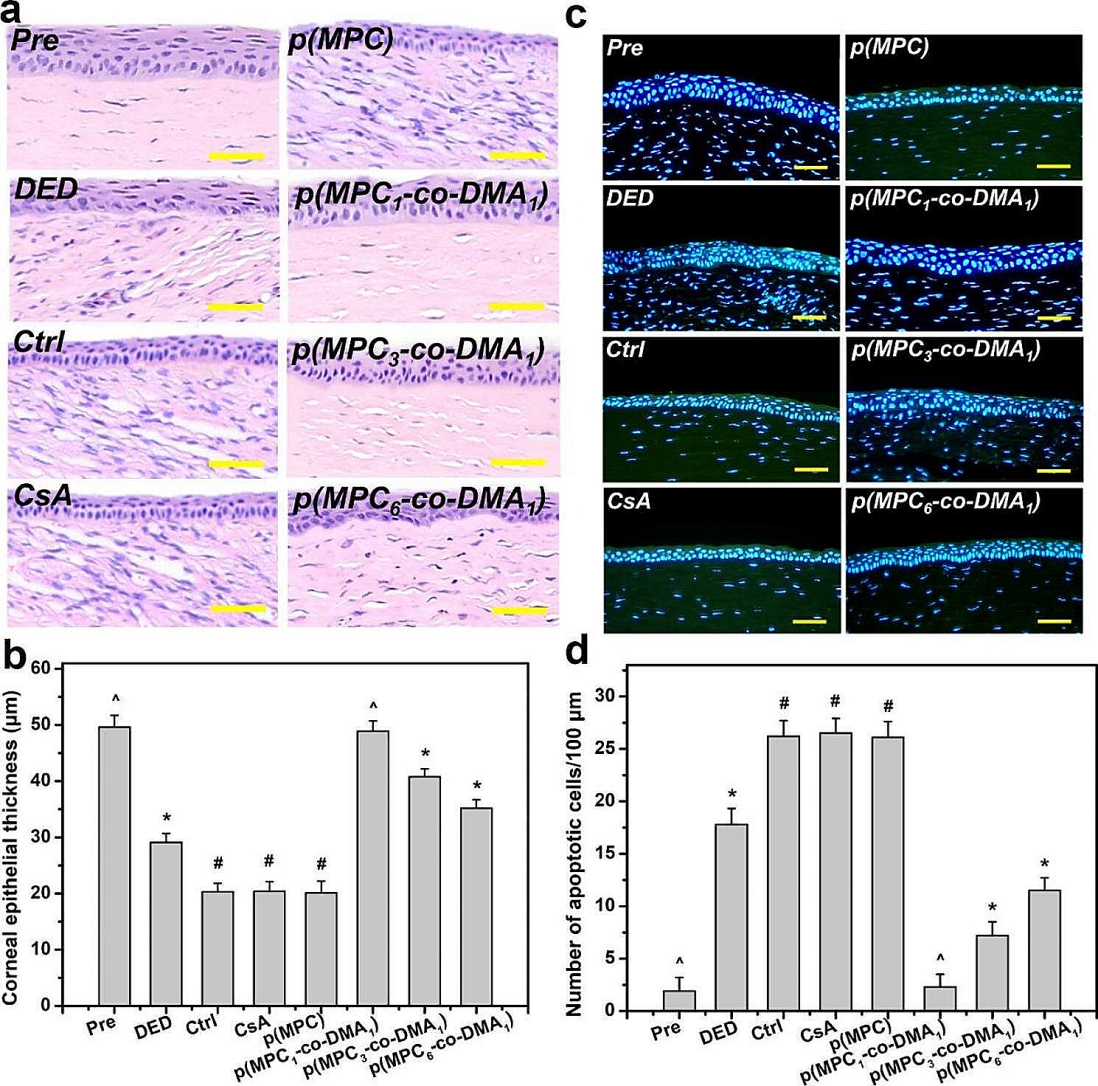
Histological assessment for corneal protection and anti-apoptosis capability. (**a**) Histological images of corneal epithelium, (**b**) corneal epithelial thickness, (**c**) relative TUNEL area of the cornea and (d) TUNEL staining mean fluorescence intensity of corneal epithelium in rabbit eyes at preoperation (Pre) and those with experimentally induced dry eye (DED) before and after 4 days of topical administration of various formulations. DED-induced animals receiving ATS without polymers and drug serve as control group (Ctrl). Scale bars: 50 μm. Values are mean ± SD (*n* = 6). *p < 0.05 vs. all groups; #*p* < 0.05 vs. Pre, DED, p(MPC_1_-*co*-DMA_1_), p(MPC_3_-*co*-DMA_1_), and p(MPC_6_-*co*-DMA_1_) groups; ^*p* < 0.05 vs. DED, Ctrl, CsA, p(MPC), p(MPC_3_-*co*-DMA_1_), and p(MPC_6_-*co*-DMA_1_) groups

**Fig. 9:**
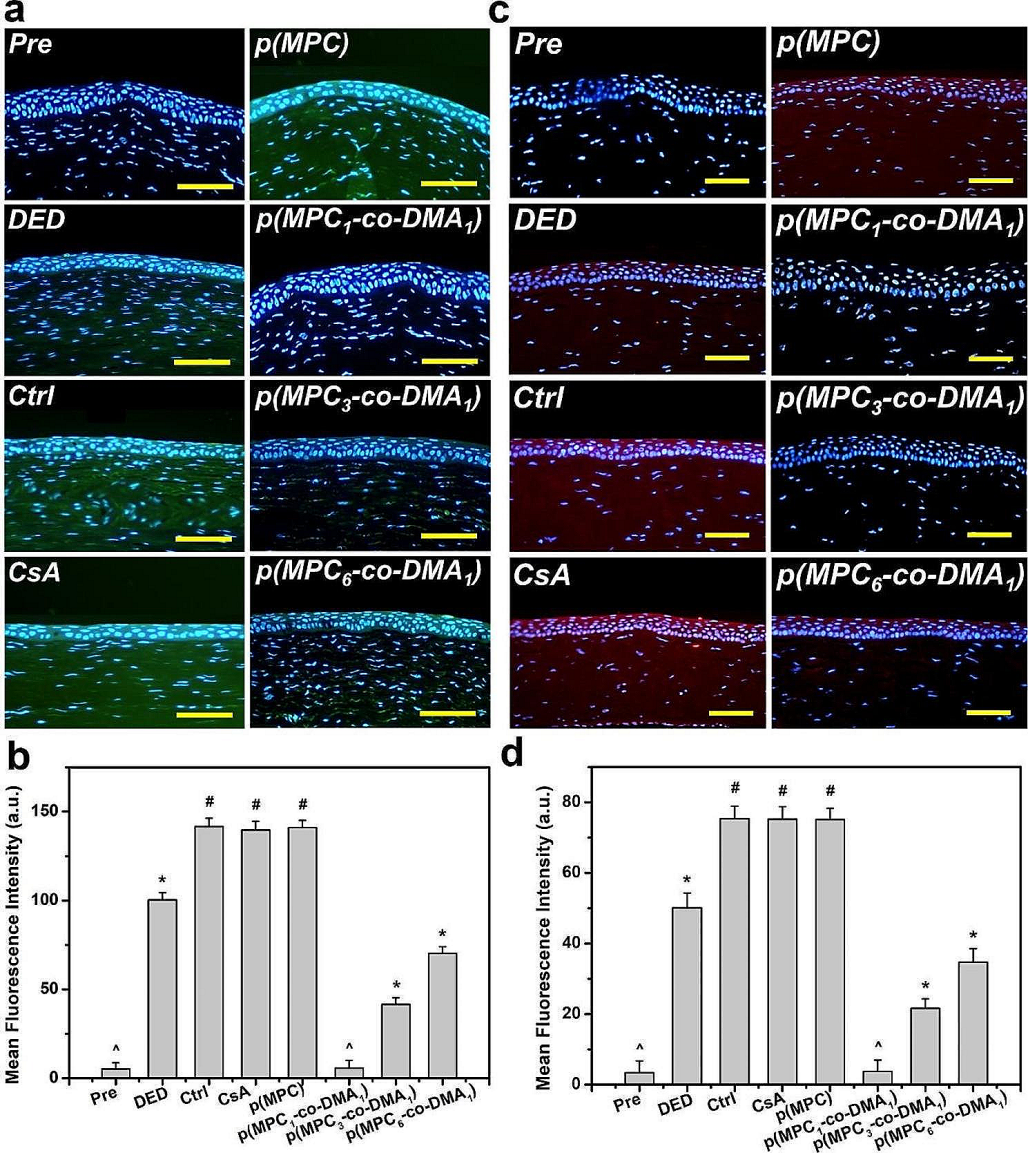
Histological assessment for antioxidation and anti-inflammation capability. (**a**) Fluorescence images and (**b**) mean fluorescence intensity of corneal epithelium in rabbit eyes and those with experimentally induced DED 4 days after various samples administration. DED animals receiving ATS without nanomaterials and drug served as control groups (Ctrl). Green fluorescence is DCFH-DA-positive staining. Scale bars: 100 μm. (**c**) IL-6 immunofluorescence staining images and (**d**) mean fluorescence intensity of corneal epithelium in rabbit eyes and those with experimentally induced DED 4 days after various samples administration. DED animals receiving ATS without nanomaterials and drug served as control groups (Ctrl). Red fluorescence is IL-6-specific antibody staining. Scale bars: 50 μm. Values are mean ± SD (*n* = 6). *p < 0.05 vs. all groups; #*p* < 0.05 vs. Pre, DED, p(MPC_1_-*co*-DMA_1_), p(MPC_3_-*co*-DMA_1_), and p(MPC_6_-*co*-DMA_1_) groups; ^*p* < 0.05 vs. DED, Ctrl, CsA, p(MPC), p(MPC_3_-*co*-DMA_1_), and p(MPC_6_-*co*-DMA_1_) groups

Tissue samples were taken at the endpoint (i.e., at 4 days postoperatively) to determine the histological changes in response to different treatments. The corneal epithelia for ATS-only treatment group (Ctrl group), CsA group, and p(MPC) group were thinner than that for the DED group (Fig. 8a). In contrast, p(MPC-*co*-DMA)-treated animals showed significantly higher tissue thicknesses (*p* < 0.05) among all groups (Fig. 8b). Interestingly, the corneal epithelial integrity for the p(MPC_1_-*co*-DMA_1_) group was evenly matching that for the Pre group. TUNEL assays were further conducted to analyze the genomic fragmentation and cell apoptosis in BAC-induced DED rabbits with topically administered medications after 4 days (Fig. 8c and d). For the DED group, the apoptotic corneal epithelial cells emitted an intense green fluorescence, indicating the relationship between inflammation and cellular apoptosis [72]. For the CsA and p(MPC) groups, the TUNEL-labeled apoptotic cells still displayed intense green dye of DNA fragments after a 4-day treatment. This strongly implies that CsA and p(MPC) were ineffective treatments for DED. However, the p(MPC-*co*-DMA) groups exhibited successful inhibition of cell apoptosis and there is a significant decrease (*p* < 0.05) in the number of apoptotic cells and in green fluorescence after 4 days of topical administration. The smaller signal for ROS fluorescence for the p(MPC-*co*-DMA) groups also shows that there is a cytoprotective effect against reactive species (Fig. 9a and b). Tear film hyper-osmolarity caused by desiccating environmental stress can up-regulate the secretion of these pro-inflammatory cytokines (e.g. IL-6 and TNF-α), subsequently triggering chronic immune-based inflammation cascade and ultimately leading to the disruption of corneal epithelial barrier [81]. As depicted in Fig. 9c and d and S16, the amounts of pro-inflammatory factors in corneal epithelial tissue after p(MPC-*co*-DMA) instillation decrease, compared to other nanoformulations. A combination of the hydration lubrication of MPC and the antioxidant action of DMA is effective in treating ocular dryness. Remarkably, all the indicators in histological studies of p(MPC_1_-*co*-DMA_1_)-treated group were comparable to that in the Pre group, which significantly supports the therapeutic efficacy of the polymer composition for DED. The results for protecting corneal cells from a hyperosmotic environment, mitigating cellular apoptosis/inflammation, and suppressing ROS production show the synergistic therapeutic effect of p(MPC-*co*-DMA).

**Fig. 10:**
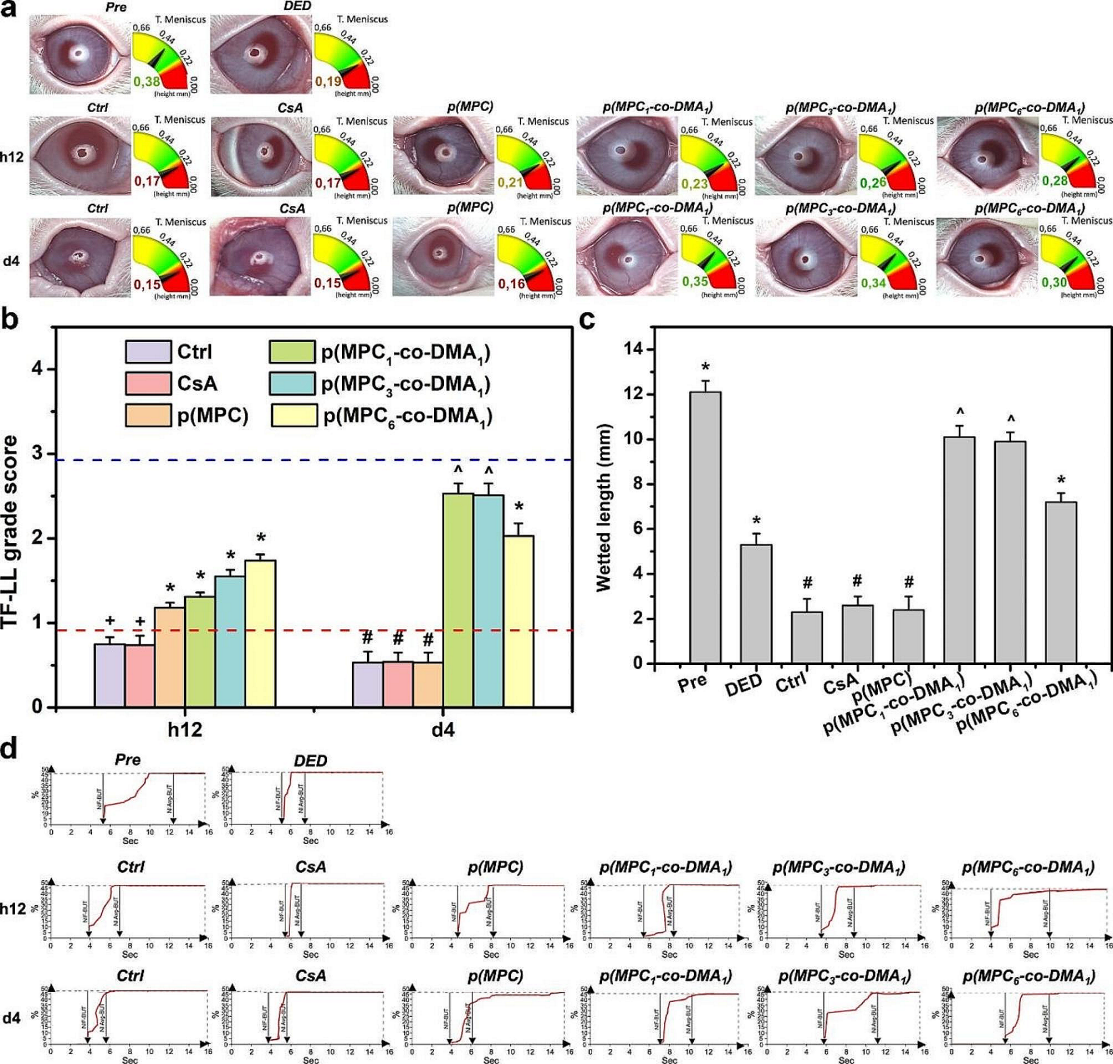
Clinical assessment of nanoformulations. (**a**) The images show tear meniscus height (TMH) measurement, lipid layer (TF-LL) pattern and (**b**) grading scale of interferometric patterns of Pre, DED, Ctrl, CsA, p(MPC), p(MPC_1_-*co*-DMA_1_), p(MPC_3_-*co*-DMA_1_), and p(MPC_6_-*co*-DMA_1_) groups at h12 and 4 days. The blue and red dash line represents the value of Pre and DED group, respectively. (**c**) The wetted length of the Schirmer paper strip for the rabbit eyes before drug administration (Pre) and those with experimentally induced DED 4 days after topical administration of CsA, p(MPC), p(MPC_1_-*co*-DMA_1_), p(MPC_3_-*co*-DMA_1_), and p(MPC_6_-*co*-DMA_1_). DED animals receiving artificial tear solution (ATS) without nanomaterial and drug serve as control groups (Ctrl). Values are mean ± SD (*n* = 6). *p < 0.05 vs. all groups; +*p* < 0.05 vs. Pre, DED, p(MPC), p(MPC_1_-*co*-DMA_1_), p(MPC_3_-*co*-DMA_1_), and p(MPC_6_-*co*-DMA_1_); #*p* < 0.05 vs. Pre, DED, p(MPC_1_-*co*-DMA_1_), p(MPC_3_-*co*-DMA_1_), and p(MPC_6_-*co*-DMA_1_); ^*p* < 0.05 vs. Pre, DED, Ctrl, CsA, p(MPC), and p(MPC_6_-*co*-DMA_1_). (**d**) The tear film first break-up time (NIF-BUT) and tear film average break-up time (NIAvg-BUT) measurements of Pre, DED, Ctrl, CsA, p(MPC), p(MPC_1_-*co*-DMA_1_), p(MPC_3_-*co*-DMA_1_), and p(MPC_6_-*co*-DMA_1_) groups at h12 and 4 days

The height of the tear meniscus and the lipid layer thickness for the tear film were measured using an ocular surface analyzer. The normal range for tear meniscus height is from 0.2 to 0.5 mm. For the DED group, the tear meniscus height in is less than 0.2 mm [82, 83]. As depicted in Fig. 10a, all polymer samples demonstrate an increase in tear volume after a h12 topical treatment. Nagai and colleagues showed that p(MPC) can enhance corneal water retention, increase tear volume, and prolong tear film break-up time without increasing the total mucin content in the tear film of a normal rabbit [25]. The tear meniscus height of p(MPC) and other copolymer groups were ≥ 0.21 mm (more prominent than Ctrl and CsA groups at 0.17 mm) at h12 postoperatively. p(MPC) is non-fouling but the zwitterionic polymer alleviates dry eye symptoms because of its lubricity, and supplements the meibomian lipid layer in precorneal tear film. Therefore, an increase in the tear film lipid layer (TF-LL) thickness supports this assumption (Fig. 10b). However, only catechol-functionalized polymers can extend the pharmacological effect after a 4-day treatment owing to their superior mucoadhesive and bioactive properties of DMA (Fig. 10c). All p(MPC-*co*-DMA) groups showed thickness ≥ 0.3 mm in the tear meniscus layer and were approaching the TF-LL score of 3 (Fig. 10a and b), suggesting the tear films were in the process of regeneration.

The tear film break-up time (BUT) is an index for direct clinical examination of the tear film, which refers to the time from the first complete blink to the appearance of the first dry spot on the tear film. The BUT index is short if it is less than 10 s, because there is insufficient tear secretion or an abnormality in the ocular surface. In clinical terms, the average rupture time for normal individuals is about 10 ∼ 13 s, but the average rupture time for DED patients is about 7 ∼ 8 s. After a h12 treatment, all formulations of MPC-containing polymer increase the average break-up time of tear film, indicating stabilization of tear osmolarity (Fig. 10d and Table S4). Remarkably, the interblink interval of rabbits extended significantly in all p(MPC-*co*-DMA) groups at 4 days postoperatively. This data confirms that catechol-functionalized polyzwitterion has a superior synergistic therapeutic effect, including hydration lubrication, supplementing of the lipid layer, mucoadhesion and anti-oxidation/anti-inflammation activity.

## Conclusions

In summary, we have successfully developed an intrinsically therapeutic nanoformulation p(MPC-*co*-DMA) via random free-radical copolymerization to the alleviation DED. In general, p(MPC-*co*-DMA) has a dual therapeutic effect: MPC portion enhances lubrication, supplements lipid layer, and relieves hyperosmotic stress on the ocular surface; while DMA residues offers self-triggering antioxidant and anti-inflammation activity. Owing to catechol moieties, two mucoadhesive mechanisms of p(MPC-*co*-DMA) were detected: (1) Tethered catechol residues on the polymer chains of p(MPC-*co*-DMA) interact with mucins via strong hydrogen bonding and covalent conjugation with amine groups via Michael addition and/or a Schiff base reaction and (2) an inter-chain interaction between adsorbed polymers and free-floating polymers can occur via catechol-catechol coupling. As a result, the copolymers could enhance their retention time and therapeutic effect on the ocular surface. By increasing the catechol portion, p(MPC-*co*-DMA) showed an improvement in anti-inflammatory and antioxidant capabilities, thereby facilitating down-regulation of pro-inflammatory factors (i.e., IL-6 and TNF-α) and cytoprotection by quenching intracellular ROS production and mitigating cell apoptosis. In a rabbit model of DED, single topical instillation of p(MPC_1_-*co*-DMA_1_) depicted clear signs of tissue restoration at 4 days postoperatively, evidenced by elevating epithelial cell recovery and refraining the endothelial cell deterioration. In clinical assessment, the administration of p(MPC_1_-*co-*DMA_1_) can improve tear fluid production about 5 times compared with the commercial CsA group. These findings strongly imply the potential therapeutic effect of the nanoformulations based entirely on synthetic biomaterials for effective DED treatment.

### Electronic supplementary material

Below is the link to the electronic supplementary material.

Supplementary Material 1

## Data Availability

No datasets were generated or analysed during the current study.
