# Supplementary material for: Mucoadhesive, antioxidant, and lubricant catechol-functionalized poly(phosphobetaine) as biomaterial nanotherapeutics for treating ocular dryness
Source: J Nanobiotechnology. 2024 Apr 8;22:160. doi: 10.1186/s12951-024-02448-x (PMC11000383; doi:10.1186/s12951-024-02448-x)
Supplement: Supplementary file 1 — Supplementary Material 1 [file 12951_2024_2448_MOESM1_ESM.docx]

Supporting information to the manuscript

**Mucoadhesive, antioxidant, and lubricant catechol-functionalized poly(phosphobetaine) as biomaterial nanotherapeutics for treating ocular dryness**

Hoang Linh Bui ^a^, Yun-Han Su ^b^, Chia-Jung Yang ^b^, Chun-Jen Huang ^c,d,e,*^, Jui-Yang Lai ^b,f,g,h,i,*^

^a^ Department of Biomedical Sciences and Engineering, National Central University, Taoyuan 32023, Taiwan.

^b^ Department of Biomedical Engineering, Chang Gung University, Taoyuan 33302, Taiwan.

^c^ Department of Chemical and Materials Engineering, National Central University, Taoyuan 32023, Taiwan.

^d^ R&D Center for Membrane Technology, Chung Yuan Christian University, Taoyuan 32023, Taiwan.

^e^ NCU-Covestro Research Center, National Central University, Taoyuan 32023, Taiwan.

^f^ Department of Ophthalmology, Chang Gung Memorial Hospital, Linkou, Taoyuan 33305, Taiwan.

^g^ Department of Materials Engineering, Ming Chi University of Technology, New Taipei City 24301, Taiwan.

^h^ Research Center for Chinese Herbal Medicine, College of Human Ecology, Chang Gung University of Science and Technology, Taoyuan 33303, Taiwan.

^i^ Center for Biomedical Engineering, Chang Gung University, Taoyuan 33302, Taiwan.

* Corresponding authors. Prof. Chun-Jen Huang, E-mail: [cjhuang@ncu.edu.tw](mailto:cjhuang@ncu.edu.tw) (C.-J. Huang); Prof. Jui-Yang Lai, E-mail: [jylai@mail.cgu.edu.tw](mailto:jylai@mail.cgu.edu.tw) (J.-Y. Lai).

**Synthesis of DMA**

DMA synthesis was adapted to previous work [1] with slight modification. 5 g of tetraborate and 2 g of sodium bicarbonate were dissolved in 50 mL of deionized (DI) water. The solution was degassed with argon gas for 20 min, followed by the addition of 2.5 g of dopamine hydrochloride powder into the prepared solution. 2.35 mL of methacrylic anhydride in 12.5 mL THF was prepared separately and added slowly to the dopamine-containing solution. 1 M NaOH was added dropwise into the reaction flask to achieve a moderately basic mixture (pH 8 or above). The reaction mixture was protected in argon gas and then stirred for 24 h. Afterward, the resulting solution was filtered. The aqueous solution was added with 25 mL of ethyl acetate and acidified with 6 M HCl to pH 2. The organic layer was isolated from the acidified solution after 1 min of phase separation, and dried by adding anhydrous MgSO_4_. Then, the solution was concentrated with a rotary evaporator. A precipitated solid was formed in the concentrated solution by stirring vigorously with hexane. The white powder was acquired by centrifuging the solution at 9000 rpm for 10 min and rinsed for three times by dissolving in 20 mL ethyl acetate and precipitating with 300 mL hexane to improve product purity. The product was dried in vacuum for 6 h and achieved production yield of 68%.

**3-(4,5-dimethylthiazol-2-yl)-5-(3-carboxymethoxyphenyl)-2-(4-sulfophenyl)-2H-tetrazolium (MTS) test**

Cells were subcultured by trypsinization at a split ratio of 1:3. The SIRC cells with a density of 5 × 10^4^ cells/well were seeded into 24 well plates by 1 mL/well. The medium was then replaced with fresh culture medium comprising different types of the various p(MPC-*co*-DMA) samples. The cells grown without the p(MPC-*co*-DMA) samples served as control (Ctrl) groups. The morphology of SIRC cells was observed by a phase-contrast microscope (Nikon, Melville, NY, USA) after the cell cultures were exposed to different types of the p(MPC-*co*-DMA) samples for 2 days. In addition, the metabolic activity was estimated by a cell viability MTS assay. The absorbance at 570 nm was determined using a microplate spectrophotometer. Data were expressed as relative MTS activity compared to that of the control. Results were averaged on four independent runs.

**Live/dead assay**

Cell viability was performed using a Live/Dead Viability/Cytotoxicity Kit (Molecular Probes, Eugene, OR, USA) followed by observations under fluorescence microscopy (Axiovert 200M; Carl Zeiss, Oberkochen, Germany), the number of live and dead cells was counted. The living cells were identified through signals of green fluorescence from the intracellular esterase activities due to cleavage of calcein AM. By contrast, a red fluorescence was produced via binding of EthD-1 to the nucleic acids in dead cells with damaged cell membranes. The viability was expressed as the average ratio of live cells to the total number of cells. Results were averaged on five independent runs.

**Comet assay**

To evaluate the genotoxicity in SIRC cells exposed to various p(MPC-*co*-DMA) samples, the comet assay was carried out according to the method described previously. After cell lysis, the slides were placed on a horizontal gel electrophoresis platform and covered with alkaline solution of 300 mM NaOH and 1 mM EDTA (pH 13). DNA was allowed to unwind for 1 h. An electrical field (300 mA, 21 V) was applied for 40 min at 4 °C. Then, the slides were stained with 4’6-diamidino-2-phenylindole (DAPI; Vector Laboratories, Peterborough, England) for visualization under a fluorescence microscope (Axiovert 200 M, Carl Zeiss, Oberkochen, Germany). Quantification of the DNA damage for each cell was made by the calculation: comet tail length (μm) = maximum total length – head diameter. For measurements, 200 cells (50 cells from each of the quadruplicate slides) were randomly selected, and the average tail lengths were determined. Results were averaged on five independent runs.

***In vivo* biocompatibility**

The modified Draize test was performed to quantify the ocular safety of materials [2]. Thirty adult New Zealand white rabbits were divided into 5 tested groups (6 animals per group), including Ctrl (ATS only), p(MPC), p(MPC_1_-*co*-DMA_1_), p(MPC_3_-*co*-DMA_1_) and p(MPC_6_-*co*-DMA_1_). A single dose of 50 µL sample was instilled into the right eye, and the left eye was untreated, which served as the control. After dose instillment, the rabbit eyes were observed for any possible adverse reactions, including irritation signs of conjunctiva, iris, and cornea before, during, and after the test. These observations were performed at regular time intervals of h12 and d4. Any ocular alteration was graded by a specific scoring system designed for this test as follows [3].

| Score | Description |
| --- | --- |
| 0 | No response |
| 1 | Weak spread erythema |
| 2 | Weak with well definite erythema |
| 3 | Sufficient erythema |
| 4 | Severe erythema combined with edema |
| 5 | Very severe erythema combined with side effects |
| 6 | Showed irritation reaction |

The biosafety of the materials was further evaluated using intraocular pressure (IOP) measurements. The IOP value was measured using a Schiotz tonometer (AMANN Ophthalmic Instruments, Liptingen, Germany), and calibrated according to the manufacturer’s instructions. Six readings were taken on each eye for each IOP determination, and the mean was calculated.

**Corneal topographer assay**

Corneal topographic maps were acquired by using the Medmont E300 Corneal Topographer (Medmont Pty Ltd., Melbourne, Australia) after 12 h and 4 days of topical instillation. Mean keratometric (K) value, displaying averaged corneal curvature at the central zone (3 mm), was extracted from topographic maps. Six independent runs were performed, and the results were interpreted as averages.

**Corneal endothelial cell density measurement**

After 12 h and 4 days of topical instillation, the morphology and density of corneal endothelial cells in rabbit eyes of all groups were characterized by specular microscopy (Topcon Optical, Tokyo, Japan). Six independent runs were performed, and the results were interpreted as averages.

**Hematoxylin and eosin (H&E) examination**

After 4 days of topical instillation, the rabbits were euthanized with CO_2_ gas for the collection of ocular tissues. The corneal specimens were fixed in 4 % paraformaldehyde in PBS, dehydrated in ethanol solutions, embedded in paraffin, and cut into 5 µm-thick sections. Subsequently, H&E staining was applied on these sections, and observed using a light microscope (Carl Zeiss). Six independent runs were performed, and the results were interpreted as averages.

**Terminal deoxynucleotidyl transferase dUTP nick end labeling (TUNEL) assay**

After 4 days of topical instillation, the rabbits were euthanized with CO_2_ gas for the collection of ocular tissues. The number of apoptotic cells was quantified using TUNEL assay (Roche Diagnostics, Indianapolis, IN, USA). Following fixation and permeabilization, the samples were incubated with a mixture of TdT solution and fluorescein isothiocyanate dUTP solution in a humidity chamber for 1 h at 37°C. Negative controls were treated with distilled water in place of TdT enzyme. Slides were counterstained with 4’,6-diamidino-2-phenylindole (DAPI; Vector, Peterborough, England, UK) for identification of cell nuclei. Under a fluorescence microscope (Carl Zeiss), the number of TUNEL-positive apoptotic cell nuclei was counted at three randomly selected fields. Six independent runs were performed, and the results were interpreted as averages.

**DCFH-DA immunofluorescence staining**

The corneal tissues were processed for oxidative stress assessment of DCFH-DA staining (10 μM). After 1 h staining and washing in phosphate-buffered saline, the slides were counterstained with DAPI (Vector) and examined under fluorescence microscope (Carl Zeiss). Six independent runs were performed, and the results were interpreted as averages.

**IL-6 and TNF-α immunofluorescence staining**

For immunofluorescence staining, sectioned corneal tissue slides were deparaffinized in xylene and then rehydrated through an ethanol series. The specimens were permeabilized and blocked overnight with 5% normal goat serum and bovine serum albumin in TBS. The specimens were then incubated with monoclonal or polyclonal antibodies against IL-6 and TNF-α (diluted 1:100) were further incubated with the appropriate Alexa Fluor 488- or 555-conjugated secondary antibody at room temperature for 1 h, and the nuclei were counterstained with DAPI (1 μg/mL). The slides were mounted with ProLong Gold, and images were captured using a Zeiss fluorescence microscope (Axio Vision 4; Carl Zeiss). Mean fluorescence intensity of corneal epithelium was measured in color histogram using the ImageJ software program. Six independent runs were performed, and the results were interpreted as averages.

**Ocular surface analysis**

(1) Tear meniscus height was measured perpendicular to the lower lid border, which corresponds to the cornea. An image focusing on the lower eyelid border was acquired. The height of the tear meniscus from the obtained image was further measured using the caliper feature in the software supplied by the manufacturer. Six separate runs were made, and the averages of the outcomes were used.

(2) The thickness of the lipid layer was measured based on optical interferometry. The interference fringes in the tear film were captured on video. Videos were further examined and compared to a collection of reference videos provided by the manufacturer. The color, reflection patterns, and motions of the lipid layer were the main evaluation criteria to obtain the thickness of tear film lipid layer. Afterward, a grading system supplied by the instrument manufacturer was used to acquire the corresponding TF-LL score. The following four-interval scale was applied: Grade 0 represents cases of almost complete nonappearance of the aqueous layer, along with lipid-contaminated mucus over the surface of the corneal epithelium; this grade was added since, without a liquid component present on the cornea it was impossible to evaluate the lipids, sometimes scattered over the ocular surface in static colored islets; grade 1 (15-30 nm) represents a faintly visible homogeneous meshwork pattern; grade 2 (31-60 nm) represents a more compact meshwork pattern with grey waves; grade 3 (61-100 nm). Six independent runs were performed, and the results were interpreted as averages.

(3) Non-invasive break-up time (NIBUT) was acquired semi-automatically by looking for aberrations in circular mires that are reflected onto the tear film using the Placido disk attachment. The first disturbance in the tear film was detected and the average break-up time for the tear film was recorded using Integrated Clinical Platform (I.C.P.) Ocular Surface Analyzer (SBM System, Turin, Italy) software. For data analysis, the first break-up time was employed. Six independent runs were performed, and the results were interpreted as averages.

**Schirmer tear test**

The tear production was evaluated via Schirmer test. Schirmer test was performed to measure the tear production using Schirmer tear test strip (Color Bar Schirmer Tear Test, EagleVision, Memphis, TN, USA) without topical anesthesia. After insertion of paper strip into the external third of the lower eyelid for 3 min, the wetted length of the strip was measured in millimeter scale. Six independent runs were performed, and the results were interpreted as averages.





**Figure S1.** ^1^H NMR spectrum of DMA monomer in DMSO-d_6_.





**Figure S2.** FTIR spectrum of DMA monomer.





**Figure S3.** ^1^H NMR spectrum of MPC monomer and p(MPC) in D_2_O.





**Figure S4.** ^1^H NMR spectra of various p(MPC-*co*-DMA) samples in D_2_O.





**Figure S5.** FTIR spectra of p(MPC) and various p(MPC-*co*-DMA) samples.





**Figure S6.** Standard calibration curve of dopamine using UV-VIS at 280 nm.


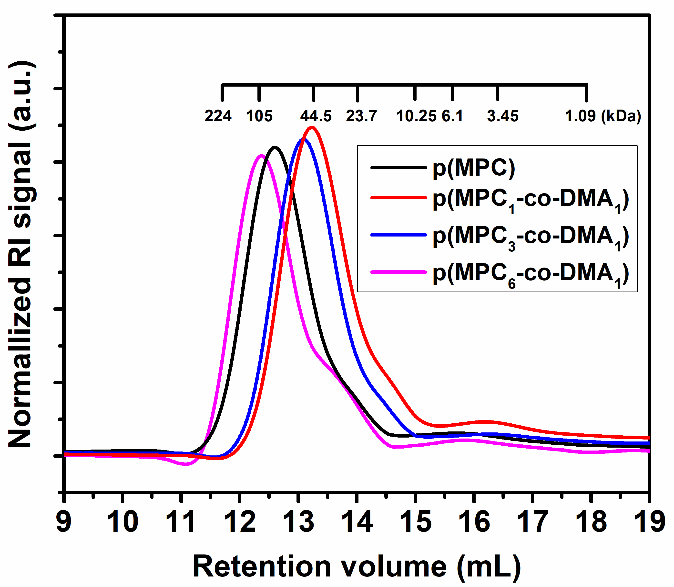


**Figure S7.** Gel-permeation chromatography (GPC) chromatograms of p(MPC) and various p(MPC-*co*-DMA) samples.


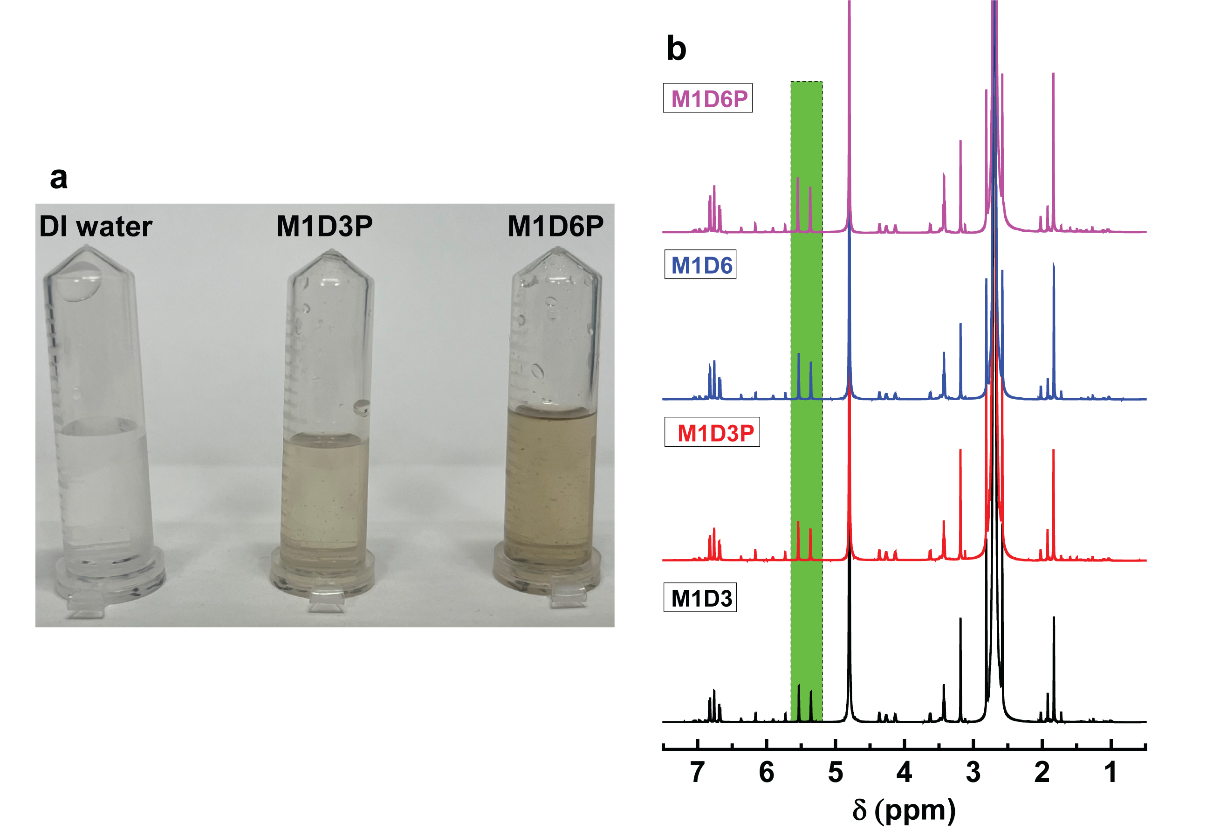


**Figure S8.** (a) Digital images of solutions containing MPC:DMA mixtures at 1:3 and 1:6 ratios after polymerization. (b) ^1^H NMR spectra of MPC:DMA mixtures at 1:3 and 1:6 before and after polymerization. The prepared solution at 1:3 and 1:6 ratios were denoted as M1D3 and M1D6, respectively. The polymerized solution were renamed as M1D3P and M1D6P, respectively.

**
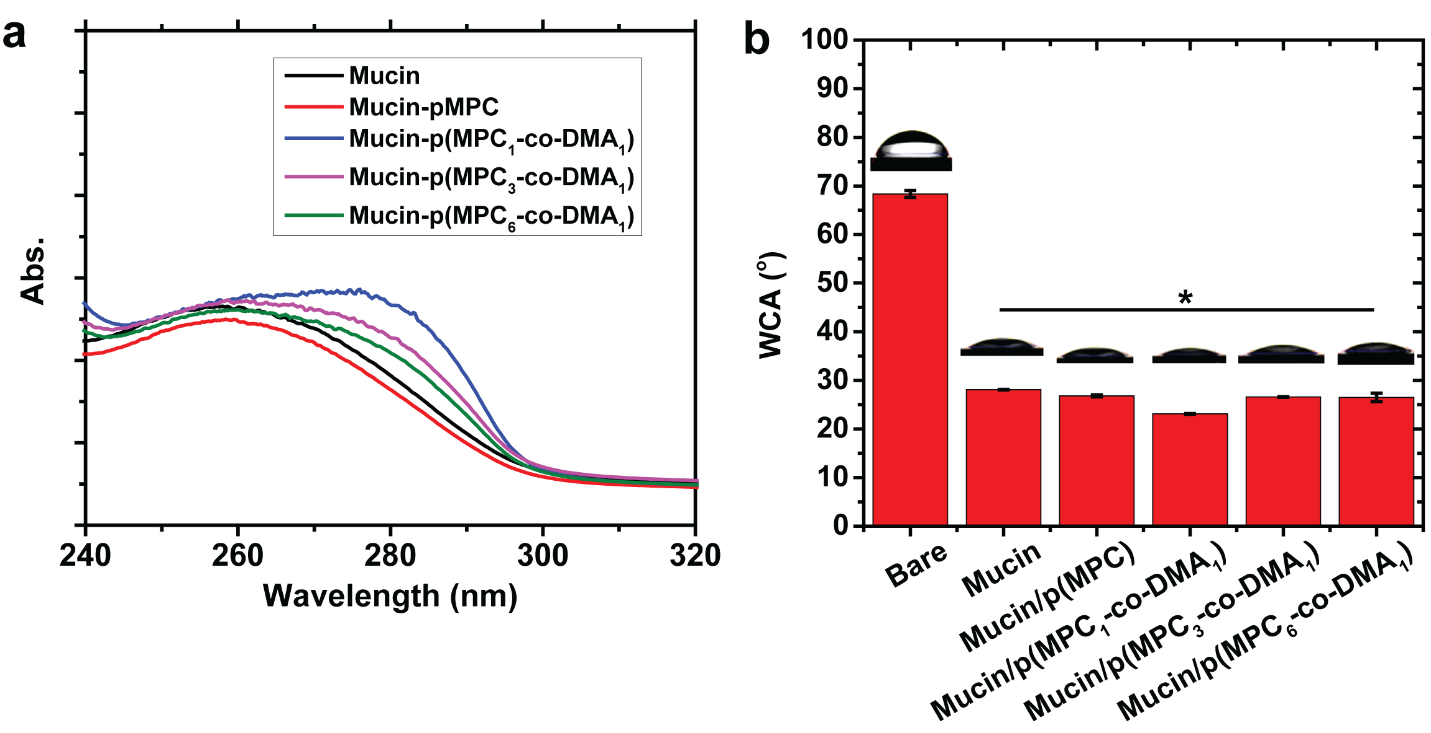
**

**Figure S9.** (a) UV-VIS spectra of mucin solution (1 wt%) mixed with synthesized polymers (1 wt%) with different compositions in PBS at pH 7.4. (b) Water contact angle (WCA) of sessile drops on silicon wafer surfaces with mucin and polymers. After mucin deposition in 1 h, mucin-coated substrates were further immersed in solution containing p(MPC), p(MPC_1_-*co*-DMA_1_), p(MPC_3_-*co*-DMA_1_) and p(MPC_6_-*co*-DMA_1_). Mucin concentration was fixed at 1 wt% and polymer solution concentration was fixed at 1 wt%. Values are mean ± SD (*n* = 3). **p* < 0.05 vs Bare.


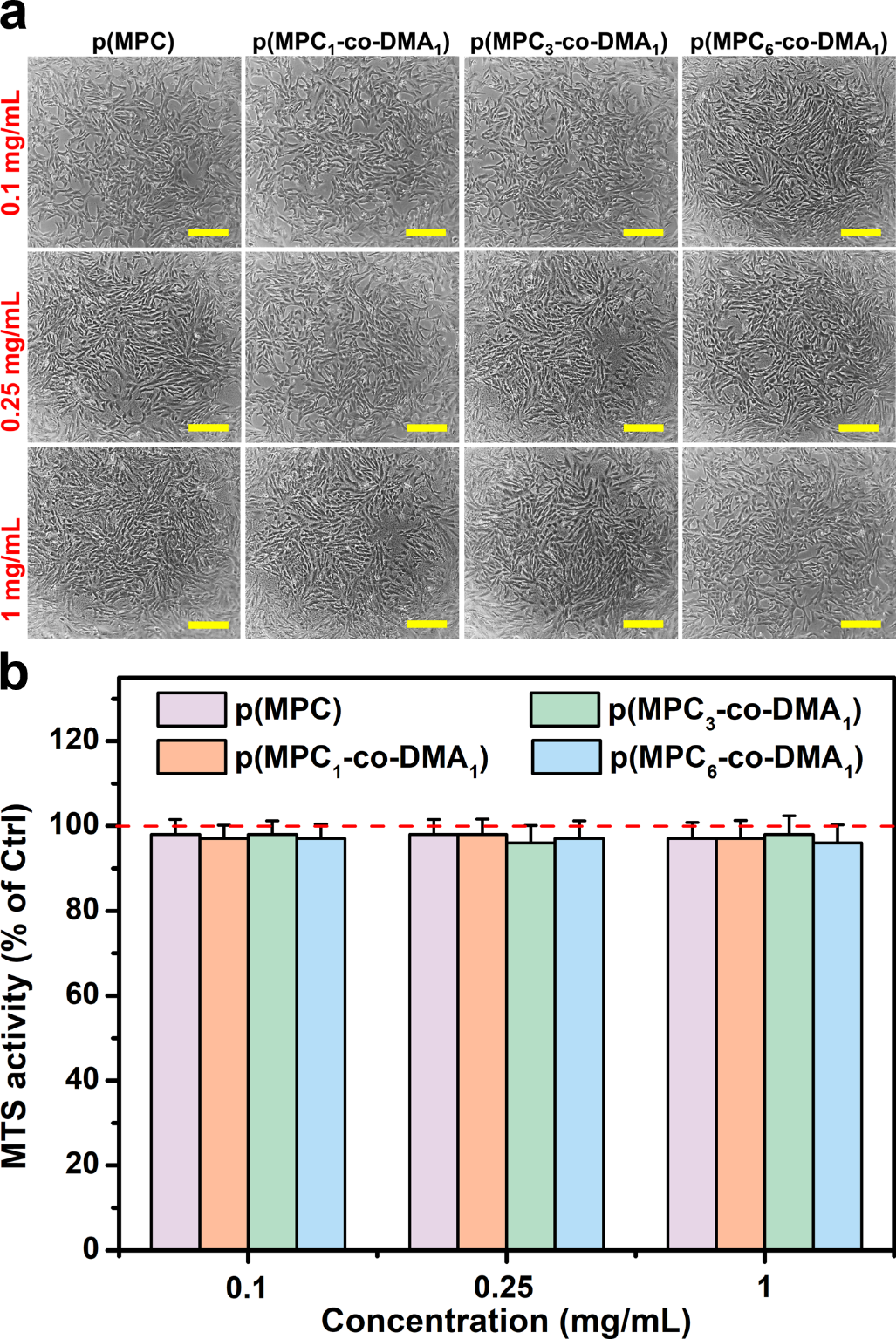


**Figure S10.** (a) Phase-contrast micrographs and (b) MTS activity of SIRC cells exposed to various p(MPC-*co*-DMA) samples for 2 -day. Ctrl: without materials. Scale bars: 100 μm. Results are expressed as percentage of Ctrl values. The red dash line represents the value of Ctrl group. Data are mean ± SD (*n* = 4).


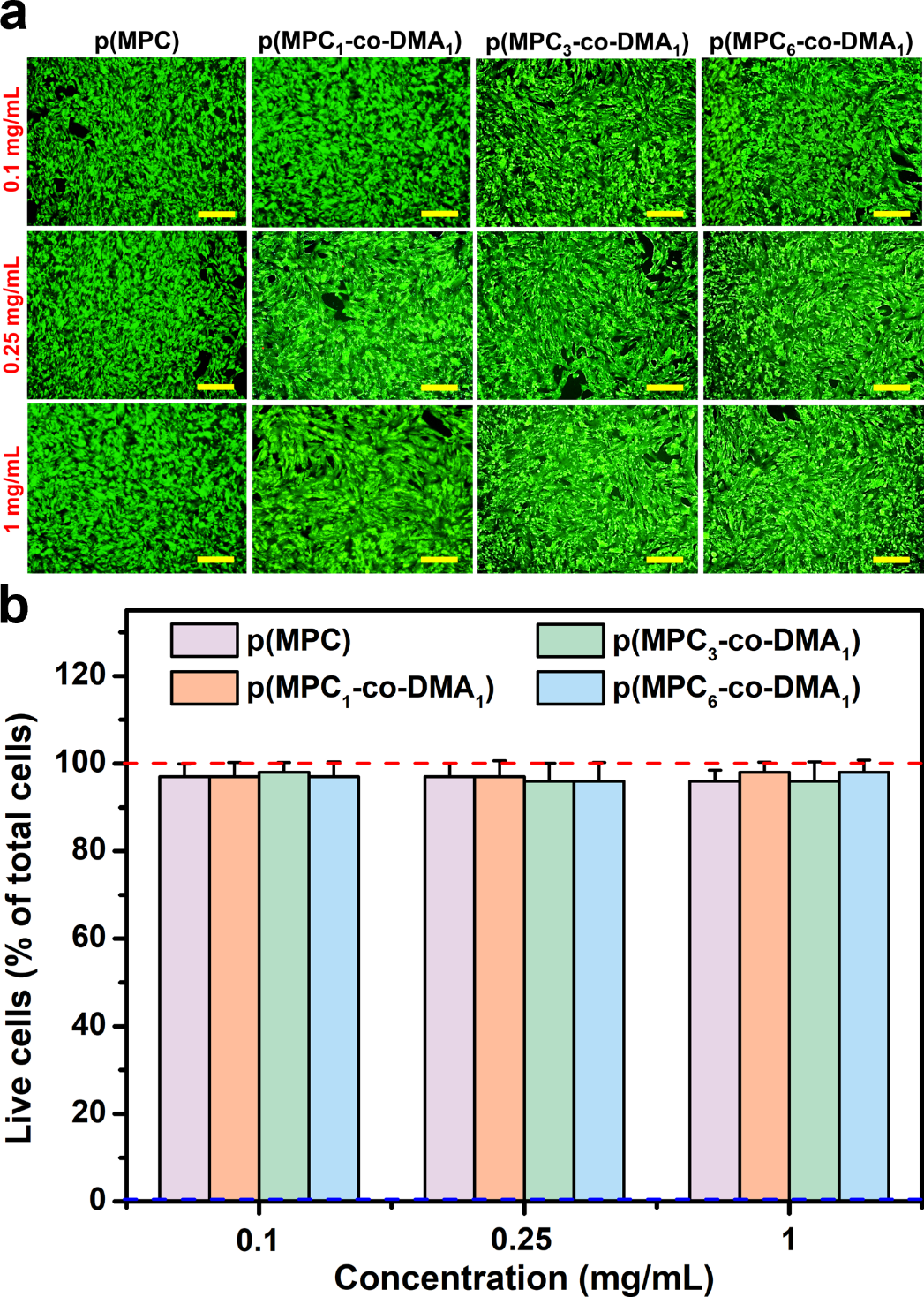


**Figure S11.** (a) Fluorescence images and (b) mean percentage of live cells of SIRC cultures after a 2-day exposure to various p(MPC-*co*-DMA) types. Ctrl: without materials. Scale bars: 100 μm. The red and blue dash lines represent the value of Ctrl and MetOH groups, respectively. Data are mean ± SD (*n* = 5).


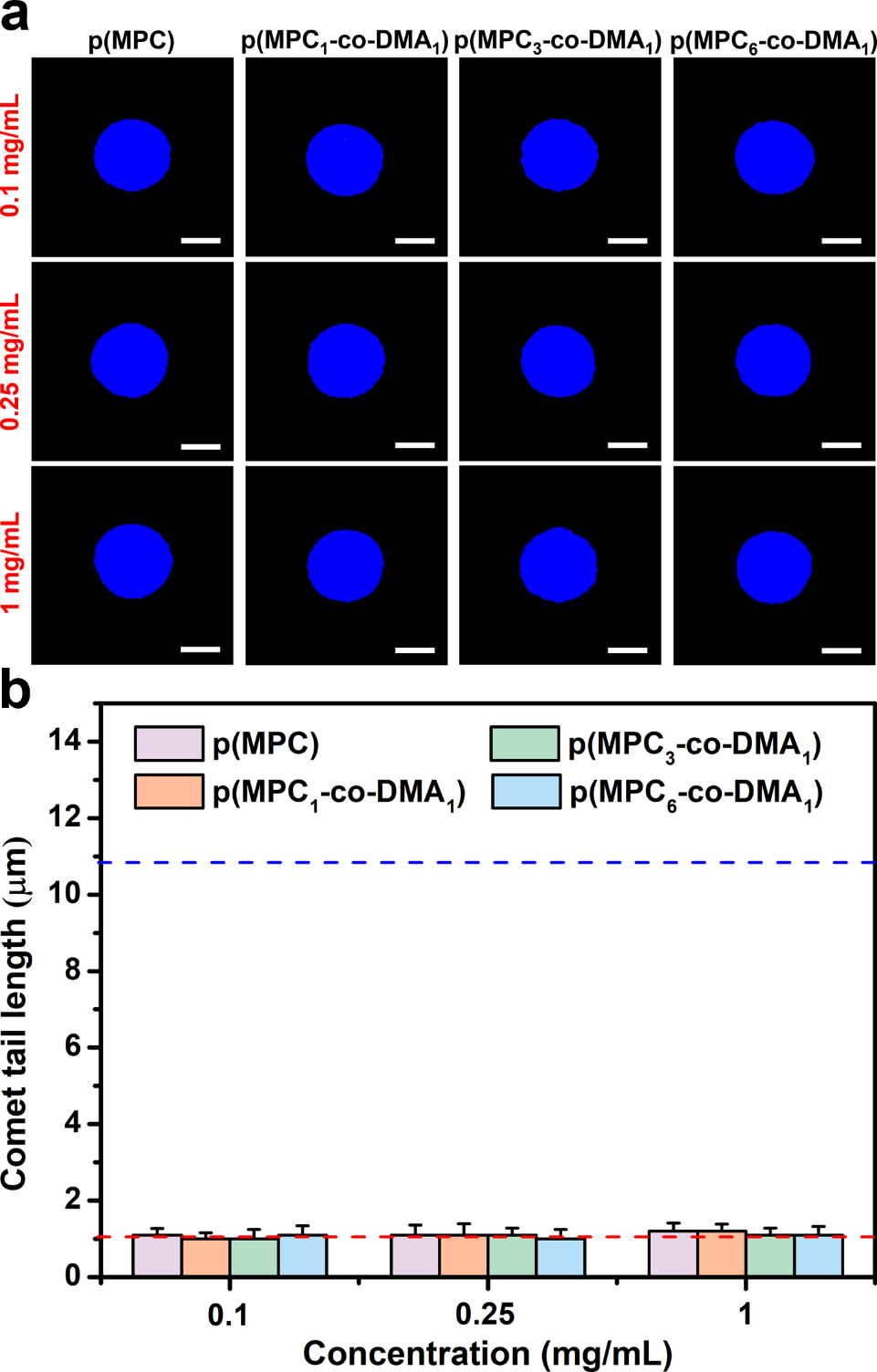


**Figure S12.** (a) Fluorescence photomicrographs and (b) tail lengths of comet assay of SIRC cultures after 2 days exposing to different p(MPC-*co*-DMA) types. Scale bars: 5 μm. The red and blue dash lines represent the value of Ctrl and Cd groups, respectively. Data are mean ± SD (*n* = 5).


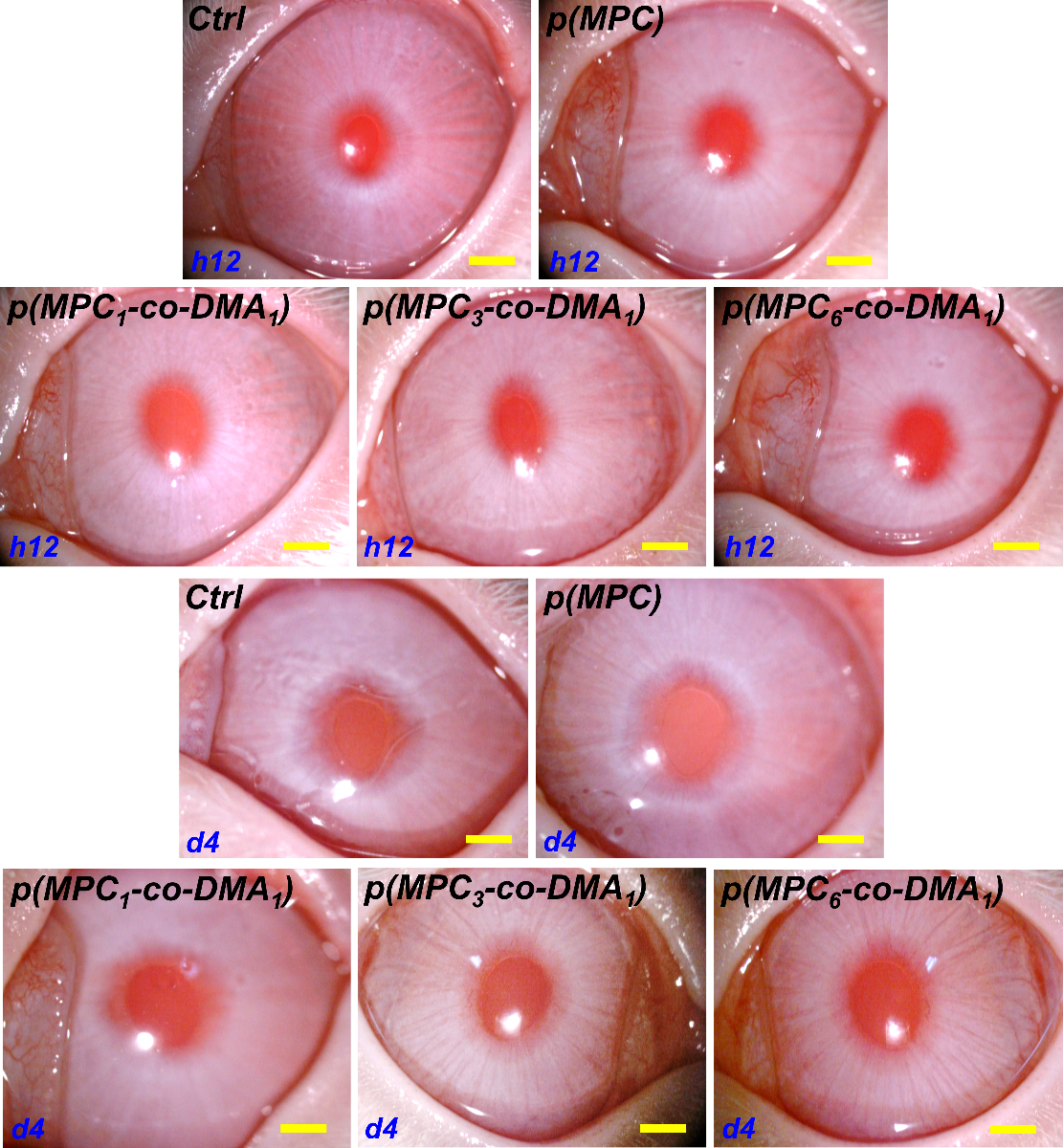


**Figure S13.** Typical slit-lamp biomicroscopic images of healthy eyes at h12 and 4 days post-instillation of polymer compositions. Scale bars: 4 mm.

**
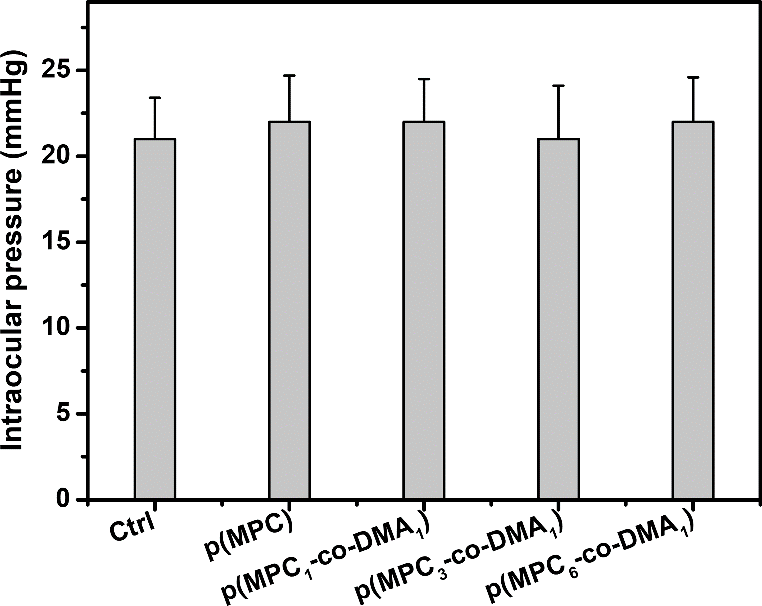
**

**Figure S14.** Measurements of intraocular pressure 4 days of p(MPC) and various p(MPC-co-DMA) samples in the ocular anterior chamber.


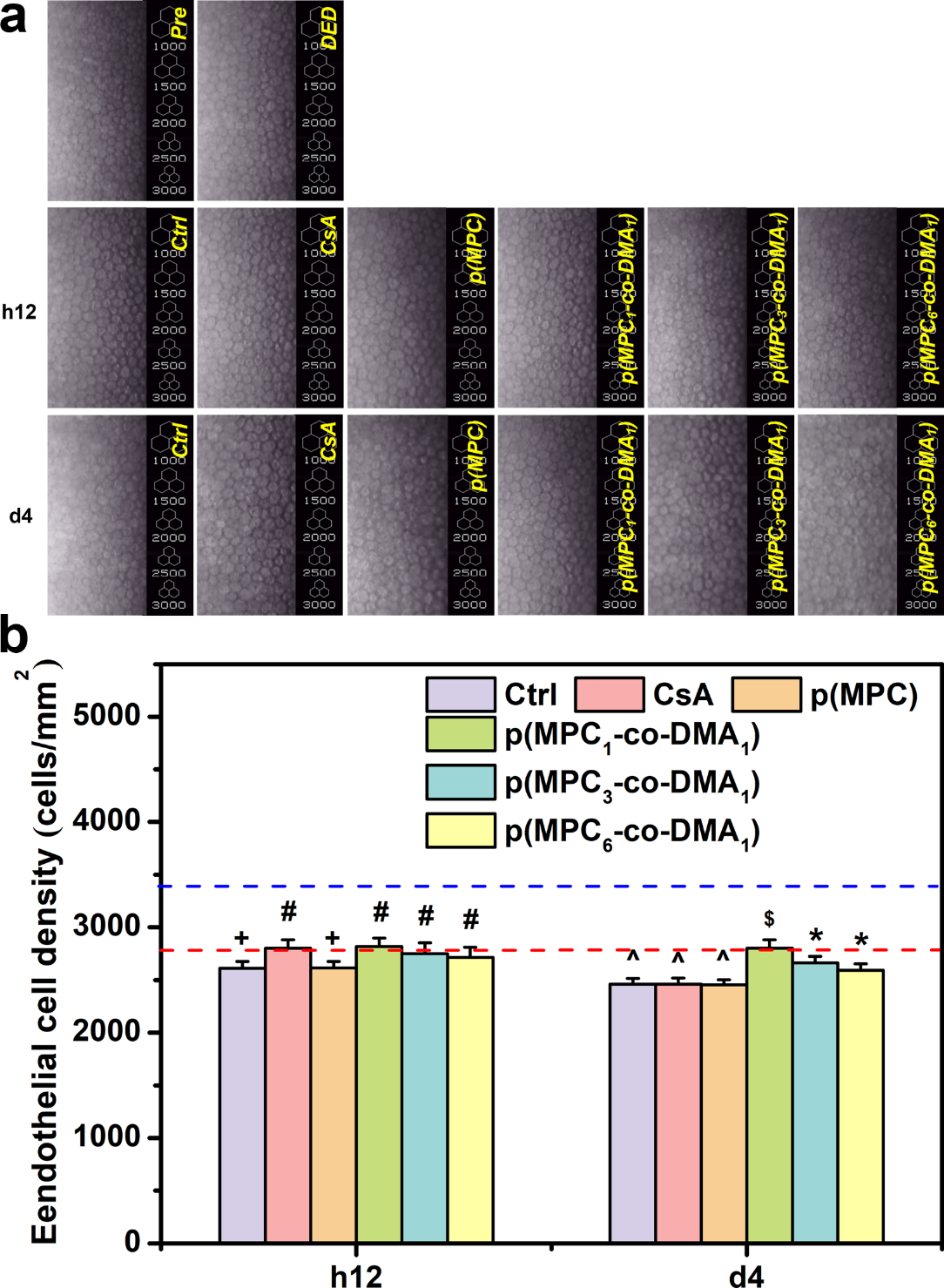


**Figure S15.** (a) Typical specular microscopic images of rabbit corneal endothelium at 12 h and 4 days after topical instillation of different formulations onto dry eyes. DED animals receiving no nanomedicine and drug serve as control groups (Ctrl). (b) Quantitative analysis of specular microscopy measurements of corneal endothelial cell density. The blue and red dash lines represent the value of Pre and DED groups, respectively. Values are mean ± SD (*n* = 6). **p* < 0.05 vs all groups; +*p* < 0.05 vs Pre, DED, CsA, p(MPC_1_-*co*-DMA_1_), p(MPC_3_-*co*-DMA_1_), and p(MPC_6_-*co*-DMA_1_) groups; #*p* < 0.05 vs Pre, Ctrl, and p(MPC) groups; ^*p* < 0.05 vs Pre, DED, p(MPC_1_-*co*-DMA_1_), p(MPC_3_-*co*-DMA_1_), and p(MPC_6_-*co*-DMA_1_) groups; $*p* < 0.05 vs Pre, Ctrl, CsA, p(MPC), p(MPC_3_-*co*-DMA_1_), and p(MPC_6_-*co*-DMA_1_) groups.


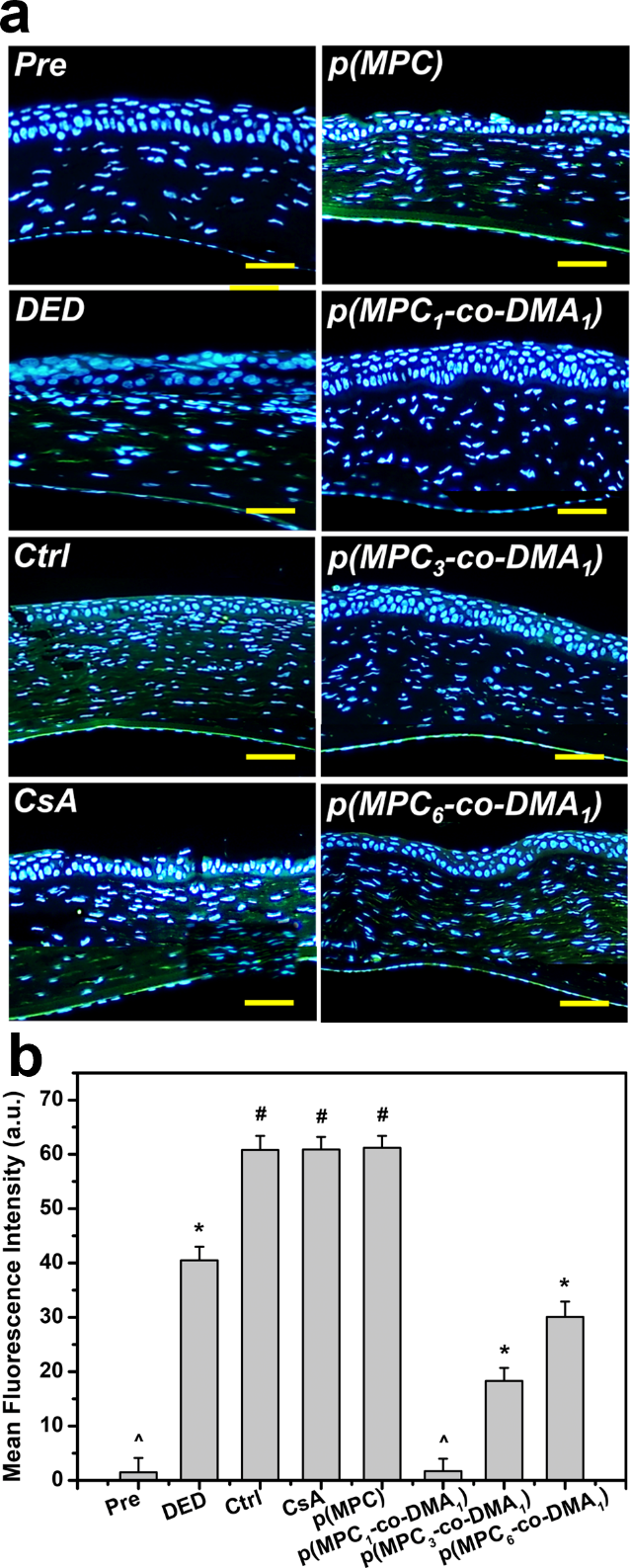


**Figure S16.** (a) TNF-α immunofluorescence staining images and (b) mean fluorescence intensity of corneal epithelium in rabbit eyes and those with experimentally induced DED 4 days after various samples administration. Green fluorescence is TNF-α specific antibody staining. Scale bars: 50 μm. DED animals receiving ATS without polymers and drug were control (Ctrl) groups. Values are mean ± SD (*n* = 6). **p* < 0.05 vs all groups; #*p* < 0.05 vs Pre, DED, p(MPC_1_-*co*-DMA_1_), p(MPC_3_-*co*-DMA_1_), and p(MPC_6_-*co*-DMA_1_) groups; ^*p* < 0.05 vs DED, Ctrl, CsA, p(MPC), p(MPC_3_-*co*-DMA_1_), and p(MPC_6_-*co*-DMA_1_) groups.

**Table S1.** Characterization of p(MPC-*co*-DMA) polymers using ^1^H NMR and GPC.

|  | **Composition (mol%) estimated from ^1^H NMR in D_2_O** | | **Molecular weight estimated from (GPC)** | **Catechol content of polymer (mg/g) recorded from UV-VIS** |
| --- | --- | --- | --- | --- |
|  | **MPC** | **DMA** | **Mn × 10^4^ (g/mol)** |  |
| **P(MPC)** | 100 | - | 9.09 | - |
| **p(MPC_1_-*co*-DMA_1_)** | 73.5 | 26.5 | 4.81 | 76.2 |
| **p(MPC_3_-*co*-DMA_1_)** | 81.8 | 18.2 | 5.57 | 46.8 |
| **p(MPC_6_-*co*-DMA_1_)** | 93.4 | 12.4 | 10.1 | 35.6 |

**Table S2.** Atomic ratio elemental analysis of the XPS.

|  | O/C | N/C | P/C |
| --- | --- | --- | --- |
| **Bare** | 2.35 | 0.03 | - |
| **Mucin** | 1.06 | 0.01 | - |
| **pMPC** | 1.02 | 0.12 | 0.07 |
| **p(MPC_1_-*co*-DMA_1_)** | 1.55 | 0.15 | 0.14 |
| **p(MPC_3_-*co*-DMA_1_)** | 0.94 | 0.10 | 0.11 |
| **p(MPC_6_-*co*-DMA_1_)** | 0.68 | 0.06 | 0.08 |

**Table S3.** Draize scores for eye irritation evaluation.

|  | **Observation at h12** | **Observation at d4** |
| --- | --- | --- |
| **Ctrl** | 0 | 0 |
| **p(MPC)** | 0 | 0 |
| **p(MPC_1_-*co*-DMA_1_)** | 0 | 0 |
| **p(MPC_3_-*co*-DMA_1_)** | 0 | 0 |
| **p(MPC_6_-*co*-DMA_1_)** | 0 | 0 |

**Table S.** Clinical evaluations and measurements of the ocular surface in animals with dry eye disease.

|  | Film average break-up time (NIAvg-BUT) (seconds) | |
| --- | --- | --- |
|  | **12 h** | **4 days** |
| Pre | 13.07 ± 1.35 | |
| DED | 8.23 ± 0.54 | |
| Ctrl | 7.64 ± 0.42 | 6.05 ± 0.76 |
| CsA | 7.49 ± 0.63 | 6.13 ± 0.54 |
| p(MPC) | 8.25 ± 0.48 | 6.11 ± 0.69 |
| p(MPC_1_-*co*-DMA_1_) | 9.06 ± 0.41 | 12.02 ± 0.58 |
| p(MPC_3_-*co*-DMA_1_) | 9.93 ± 0.35 | 12.14 ± 0.65 |
| p(MPC_6_-*co*-DMA_1_) | 10.21 ± 0.52 | 11.06 ± 0.53 |

**References**

1. Xu LQ, Pranantyo D, Ng YX, Teo SLM, Neoh KG, Kang ET, Fu GD. Antifouling coatings of catecholamine copolymers on stainless steel. Ind. Eng. Chem. Res. 2015;54:5959-67.
2. Bozdağ S, Gümüş K, Gümüş Ö, Ünlü Ö. Formulation and in vitro evaluation of cysteamine hydrochloride viscous solutions for the treatment of corneal cystinosis. Eur. J. Pharm. Biopharm. 2008;70:260-69.
3. Sayed S, Abdel-Moteleb M, Amin MM, Khowessah OM. Cubogel as potential platform for glaucoma management. Drug Deliv. 2021;28:293-305.
